# A 4,4′,4″-nitrilotriphenoxyl radical derived from Yang's biradical

**DOI:** 10.1039/d5sc06789h

**Published:** 2025-11-27

**Authors:** Qiong-Yan Hong, Bin Huang, Yanfei Niu, Cuihong Wang, Xiao-Li Zhao, Hai-Bo Yang, Xueliang Shi

**Affiliations:** a State Key Laboratory of Petroleum Molecular & Process Engineering, Shanghai Key Laboratory of Green Chemistry and Chemical Processes, School of Chemistry and Molecular Engineering, East China Normal University Shanghai 200062 China xlshi@chem.ecnu.edu.cn

## Abstract

Phenoxyl radicals have attracted considerable attention due to their unique electronic structures and wide-ranging applications in physics, chemistry, biology, and materials science. Herein, we report the synthesis and in-depth characterization of a previously unreported 4,4′,4″-nitrilotriphenoxyl radical 3, structurally derived from Yang's biradical scaffold. Interestingly, electronic structure analyses reveal that 3 is a monoradical with an open-shell doublet ground state, wherein the unpaired electron is delocalized over the three peripheral 2,6-di-*tert*-butylphenoxyl units. Notably, radical 3 is metastable in solution, reverting to closed-shell hydroxyl precursor 2 with a half-life of ∼116 minutes. The hydroxyl 2 can be deprotonated to form anionic 4, which exhibits a closed-shell singlet ground state. Their unique electronic structures are further elucidated by single-crystal X-ray diffraction and assisted by computational methods, in which 3 is fully conjugated and adopts a *C*_3_-symmetric geometry, while both 2 and 4 exhibit distinct quinoidal character with apparent *C*_2_-symmetry. These findings offer fundamental insights into the electronic structures of nitrogen-bridged polyphenoxyl radicals and establish a new design platform for stable organic open-shell systems.

## Abstract 2

Herein, we report the synthesis and characterization of a 4,4′,4″-nitrilotriphenoxyl radical 3 derived from Yang's biradical. Unlike the triplet Yang's biradical, 3 is a monoradical with a doublet ground state and a short half-life of 116 min.

## Graphical abstract

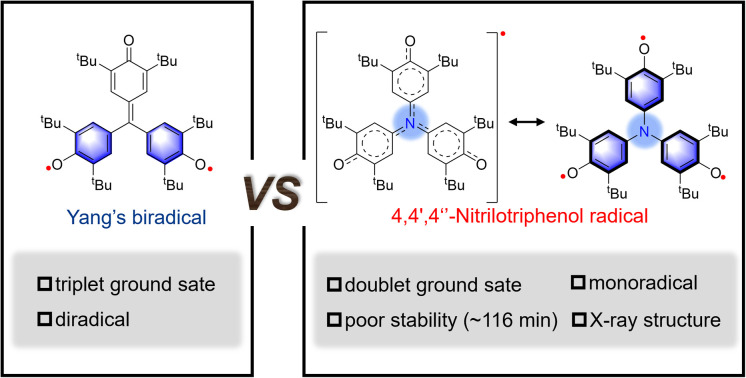

## Introduction

Phenoxyl radicals represent a unique class of highly reactive open-shell species characterized by delocalized unpaired electrons and distinctive electronic structures.^1–6^ These features underpin their widespread utility across a variety of fields, including biological systems,^7,8^ medicinal chemistry,^9,10^ organic synthesis,^11,12^ dynamic covalent chemistry,^13–15^ coordination chemistry^16^ and organic radical batteries.^17–19^ In biological contexts, phenoxyl radicals are involved in critical processes such as enzymatic catalysis^20^ and redox signaling.^21^ In medicine, their ability to scavenge reactive oxygen species (ROS) has attracted attention for therapeutic applications targeting aging-related diseases,^22^ cancer,^23^ and neurodegenerative disorders.^24^ In organic synthesis, electronic coupling between phenoxyl radicals and radical traps facilitates novel bond-forming strategies,^25,26^ while their reversible radical–radical interactions provide a foundation for dynamic covalent chemistry and the construction of complex molecular architectures and chromic materials.^13,27–29^ Moreover, their capacity to engage in electron transfer with metal centers enables the formation of redox-active coordination complexes with tunable properties.^30–32^ Additionally, phenoxyl radicals have been applied in organic radical batteries owing to their excellent redox activity.^33–36^ Importantly, these diverse functions and applications are fundamentally rooted in the electronic structures of phenoxyl radicals. Thus, a deep understanding of their electronic configurations is essential for guiding the rational design of phenoxyl-based functional materials and elucidating their structure–property–function relationships.

**Fig. 1:**
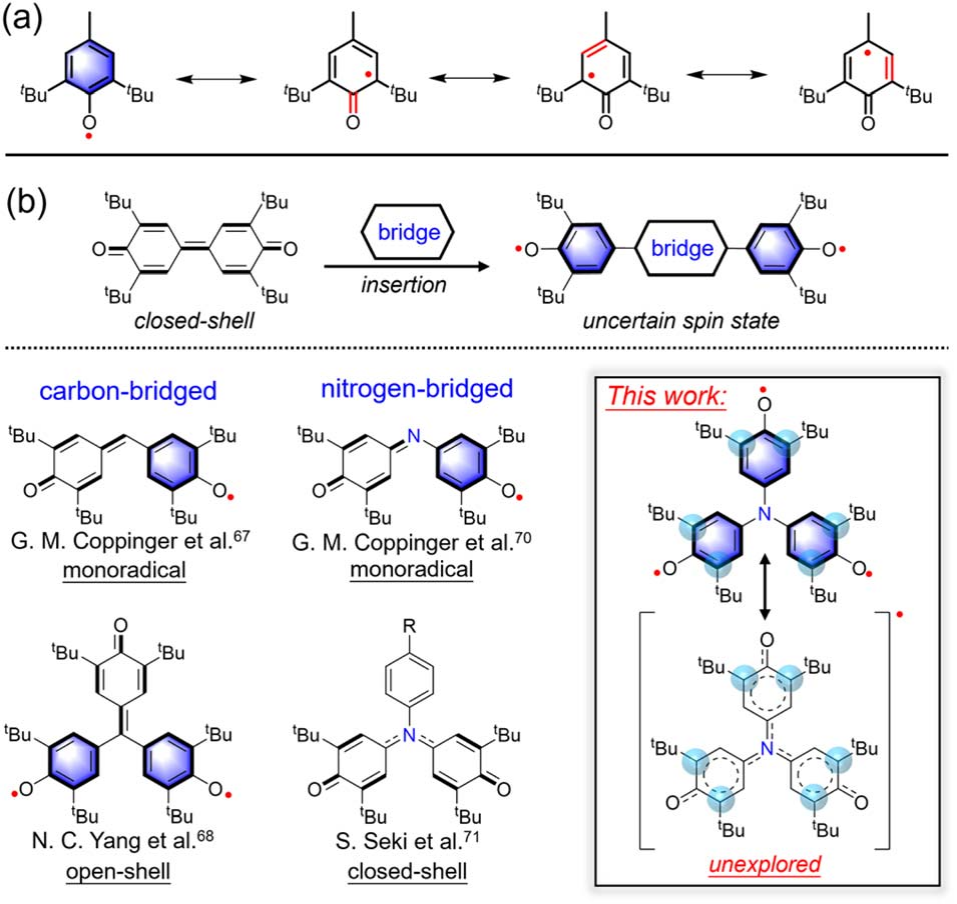
Phenoxyl radical and its substituted single atom-bridged derivatives. (a) Canonical resonance structure of phenoxyl radical. (b) Carbon-bridged and nitrogen-bridged diphenoxyl radicals, as well as 4,4′,4″-nitrilotriphenol radical in this work.

The unpaired electron in a phenoxyl radical is primarily localized on the oxygen atom and the *ortho* and *para* positions of the aromatic ring (Fig. 1a). Consequently, introducing bulky *tert*-butyl groups at these high-spin-density sites serves as an effective strategy for stabilizing the radical center.^37,38^ Beyond steric protection, the electronic delocalization of phenoxyl radicals can be finely tuned through *para*-substitution, allowing for systematic structural modifications.^39–48^ Notably, *para*-bridging enables the construction of poly(phenoxyl) radicals by linking multiple radical units, offering a valuable platform for investigating their collective electronic properties and reactivities.^1,39–54^ A diverse array of poly(phenoxyl) radicals featuring different bridging motifs has been synthesized, many of which exhibit distinctive electronic structures, pronounced near-infrared (NIR) absorption,^55^ and remarkable thermal and redox stability.^56–58^ The bridging units in these systems can generally be classified into two categories (Fig. 1b): (1) π-conjugated frameworks, including naphthalene diimide (NDI),^59^ quinoidal bisanthene,^60^ thiophene-based heterophenoquinones,^61^ perylene diimide (PDI),^62^ helicenes,^63^ boron dipyrromethene (BODIPY),^64^ and carbazole,^65^ and (2) substituted single atoms, such as methine carbon or nitrogen. The nature of the bridging motif plays a pivotal role in determining the ground-state electronic configuration. For instance, bridging *via* a methine carbon leads to the formation of the classic Galvinoxyl monoradical,^66,67^ while further adding a 2,6-di-*tert*-butylphenoxyl unit yields Yang's biradical (Fig. 1b).^68,69^ Of particular interest are nitrogen-bridged polyphenoxyl systems, which continue to attract attention due to their structural diversity and unique electronic behavior. Imine-bridged derivatives typically yield monoradicals,^70^ whereas amine linkages favor closed-shell configurations.^71,72^ Intriguingly, theoretical studies have suggested that nitrogen-centered analogs of Yang's biradical may exist as either open-shell monoradicals or high-spin quartet species. However, previously reported analogs appear unstable, and their detailed electronic structures remain poorly understood.^73,74^

In this study, we report the successful synthesis and comprehensive characterization of a previously unknown 4,4′,4″-nitrilotriphenoxyl radical 3, structurally derived from Yang's biradical framework (Fig. 1b). Single-crystal X-ray diffraction reveals that 3 adopts a planar, *C*_3_-symmetric geometry. Notably, 3 exhibits high reactivity and limited persistence in solution, with a measured half-life of ∼116 minutes in toluene under ambient conditions, gradually converting to its closed-shell hydroxyl precursor 2. Electronic structure analysis *via* DFT calculations shows that the unpaired electron in 3 is primarily delocalized over the three phenoxyl moieties, while the central nitrogen atom remains spin-inactive, consistent with the absence of ^14^N hyperfine splitting in the EPR spectrum. Variable-temperature EPR spectroscopy further confirms that 3 adopts an open-shell doublet ground state. In contrast, both 2 and its deprotonated anionic form 4, exhibit *C*_2_-symmetric geometries and are EPR silent with well-resolved ^1^H NMR signals, indicating closed-shell singlet ground states. These findings collectively reveal distinct ground-state electronic structures across this series and provide rare insights into the structure–property relationships of nitrogen-bridged polyphenoxyl radicals, offering a valuable platform for designing new open-shell organic materials.

## Results and discussion

**Fig. 2:**
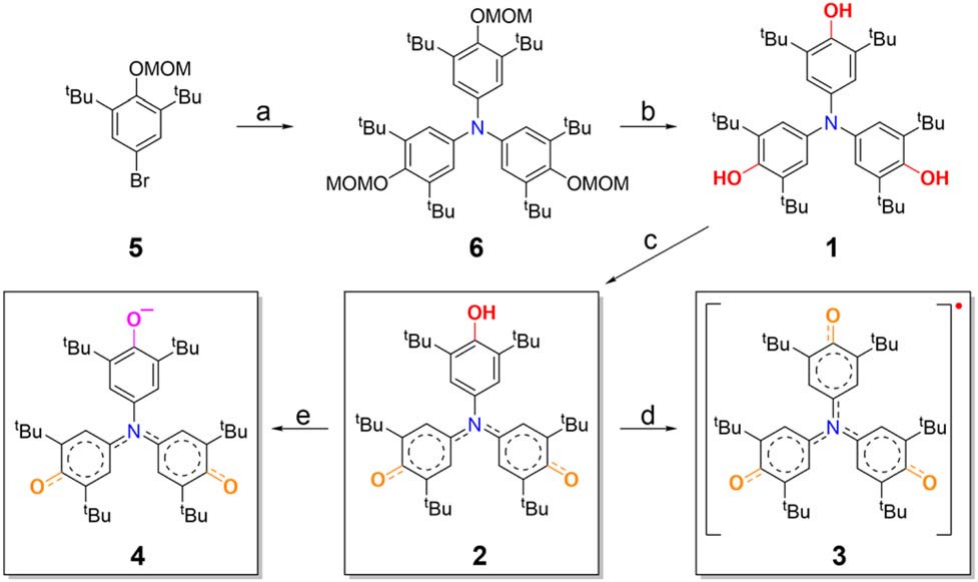
Synthetic route to 1, 2, 3 and 4. (a) Urea, TBPBF_4_, *t*-BuONa, Pd_2_(dba)_3_, 1,4-dioxane, 100 °C, yield: 77%. (b) TFA, DCM, r.t., yield: 80%. (c) K_3_[Fe(CN)_6_], NaOH, toluene, r.t., yield: 71%. (d) PbO_2_, toluene, r.t., yield: nearly quantitative. (e) TBAH, toluene, r.t., yield: nearly quantitative. TBPBF_4_: tri-*tert*-butylphosphine tetrafluoroborate; TFA: trifluoroacetic acid; TBAH: tetrabutylammonium hydroxide.

The synthesis of compounds 1–4 is outlined in Fig. 2. Inspired by the structures of Galvinoxyl radical and Yang's biradical, we introduced bulky *tert*-butyl groups at the *ortho* positions of the phenyl rings to enhance the molecular stability (Fig. 1b). The key intermediate 1, bearing three hydroxyl groups, was prepared *via* a palladium-catalyzed amination between urea and MOM-protected 2,6-di-*tert*-butyl-4-bromophenol, followed by acid-mediated deprotection of the MOM groups, affording the product in good overall yield (see Section 2 of the SI). Oxidation of 1 with potassium ferricyanide (K_3_[Fe(CN)_6_]) in a biphasic toluene/aqueous NaOH system afforded the monohydroxylated precursor 2 as a blue-violet powder in 71% yield. Further oxidative dehydrogenation of 2 using PbO_2_ gave the target radical 3 as a deep blue solid in nearly quantitative yield. Additionally, the anionic species 4 was generated by deprotonating 2 with tetrabutylammonium hydroxide (TBAH). All compounds (1–4) were fully characterized by ^1^H and ^13^C NMR spectroscopy as well as high-resolution mass spectrometry (HRMS). Specifically, the main structural difference among compounds 1–4, particularly the number of hydroxyl groups, was confirmed by Fourier-transform infrared (FT-IR) spectroscopy. 1 showed the strongest O–H stretching vibration, followed by 2, while 3 and 4 exhibit no such signal (Fig. S14). In particular, compound 3 shows an unusually low-frequency and weak C=O stretching band, consistent with its more delocalized electronic structure and elongated C–O bonds, which impart greater single-bond character and thus a reduced stretching force constant compared with 2 and 4 (Fig. S14). Crucially, their molecular structures were unambiguously confirmed by single-crystal X-ray diffraction (see below), providing solid structural evidence for their identities.

**Fig. 3:**
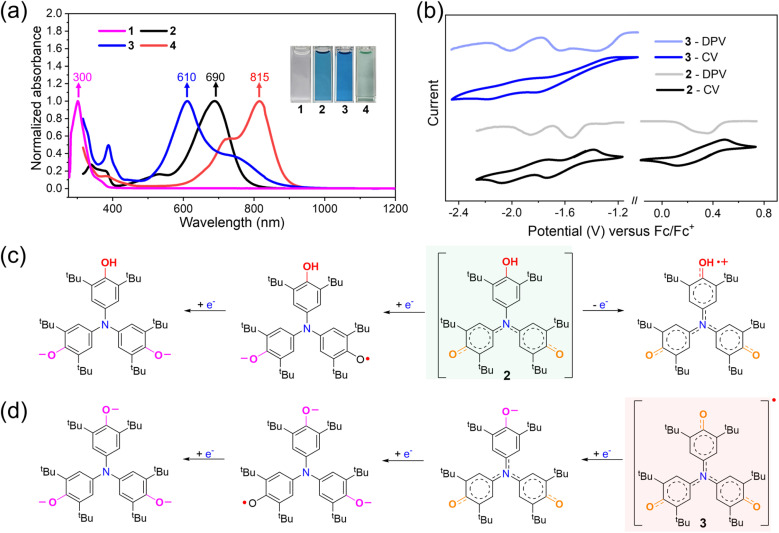
Studies of photophysical and electrochemical properties. (a) Normalized UV-vis-NIR absorption spectra (∼4.4 × 10^−4^ M in toluene) of 1, 2, 3 and 4. Insets show the photos of solutions of 1, 2, 3 and 4. (b) Cyclic voltammograms and differential pulse voltammograms of 2 and 3 in dry solution (DCM/toluene = 1/4, v/v) with 0.1 M Bu_4_NPF_6_ as the supporting electrolyte, Ag/AgCl as the reference electrode, a Pt wire as the counter electrode and a scan rate at 20 mV s^−1^. (c) The proposed redox process of 2. (d) The proposed reduction process of 3.

The photophysical properties of compounds 1–4 reveal their distinct electronic structures (Fig. 3a). The colorless compound 1 shows a strong absorption band between 260 and 400 nm, with a maximum at 300 nm. Upon oxidation to form 2, the solution turns blue, and the absorption spectrum undergoes a bathochromic shift, displaying a new peak at 690 nm. Further oxidation to the radical 3 deepens the solution color to dark blue and causes a hypsochromic shift in the absorption maximum to 610 nm, approximately 80 nm blue-shifted compared to 2. This shift is attributed to a larger SOMO–SUMO gap in 3 (2.14 eV, Fig. S33) relative to the HOMO–LUMO gap in 2 (1.93 eV, Fig. S31), as revealed by DFT calculations. Subsequent deprotonation of 2 to the anionic species 4 results in a light green solution, with a significantly red-shifted absorption maximum at 815 nm. This pronounced bathochromic shift reflects a narrower HOMO–LUMO gap in 4 (1.52 eV, Fig. S34). The calculated absorption spectra exhibit good agreement with the experimental results, particularly in the main absorption regions. Specifically, the major absorption bands of compound 2 (600–800 nm) and compound 3 (500–800 nm) mainly originate from the HOMO → LUMO transitions (Tables S3–S5 and Fig. S37). Differently, the main absorption band of compound 4 (600–900 nm) is attributed to both HOMO → LUMO and HOMO−2 → LUMO transitions (Table S6). Altogether, the clear spectral differences among 1–4 underscore their distinct electronic configurations, spanning closed-shell neutral, radical, and anionic states. These results highlight the tunability of the optical and electronic properties *via* redox and deprotonation modulation within this nitrogen-bridged triphenoxyl system.

The electrochemical properties of 2 and 3 were investigated using cyclic voltammetry (CV) and differential pulse voltammetry (DPV) in a dry DCM/toluene (1/4, v/v) solution. Due to the presence of a quinoidal conjugation electronic structure, 2 exhibited two reversible one-electron reduction waves with *E*_1/2_ at −1.86 and −1.56 V (*vs.* Fc/Fc^+^) (Fig. 3b and S25). These processes generated radical anions and dianions (Fig. 3c), while a reversible oxidation (*E*_1/2_ = 0.37 V *vs.* Fc/Fc^+^) yielded a radical cation (Fig. 3b, c and S25). Such redox behavior of 2 was consistent with that of the amine-bridged derivative of the Galvinoxyl radical 2-OMe (Fig. S24).^71^ Compared to 2, 3 displayed three reversible one-electron reduction processes with half-wave potentials (*E*_1/2_) at −2.01, −1.62, and −1.30 V (*vs.* Fc/Fc^+^) (Fig. 2b), corresponding to the formation of anions, radical dianions and trianions, respectively (Fig. 2d). No oxidation peak was observed for 3, indicating that it is a fully oxidized end state (Fig. S27). The divergent redox behaviors observed in compounds 2 and 3 reflected their fundamentally distinct electronic structures.

**Fig. 4:**
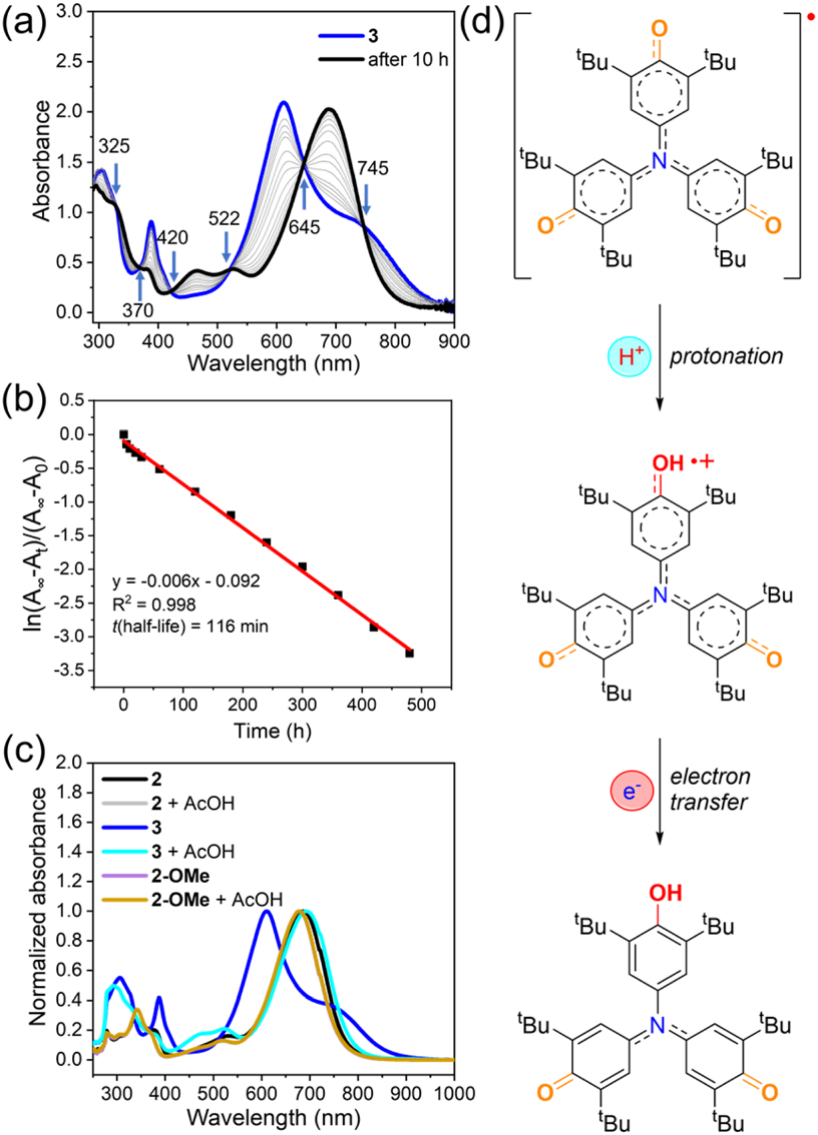
The decay process of 3 and its mechanism. (a) Change in the UV-vis-NIR absorption spectra of 3 (∼4.4 × 10^−4^ M in toluene) over time. (b) Stability plot of 3. All spectra were recorded in toluene under ambient conditions (air, room temperature, and normal light). (c) Normalized UV-vis-NIR absorption spectra (∼4.4 × 10^−4^ M in toluene) of 2, 3, and the reported compound 2-OMe^71^ before and after adding AcOH. (d) Proposed mechanism for the decay of 3 into 2 involving a protonation-coupled reduction process.

Under ambient conditions in dry toluene, 3 gradually decayed, as monitored by time-dependent UV-vis-NIR spectroscopy (Fig. 4a). Its characteristic absorption bands (500–900 nm, *λ*_max_ = 610 nm) diminished over time, while a new band (400–800 nm, *λ*_max_ = 690 nm) appeared, with well-defined isosbestic points at 325, 370, 420, 522, 645, and 745 nm, indicating a clean single-step conversion. Kinetic analysis gave a half-life of 116 minutes, confirming the meta-stable nature of 3 (Fig. 3b). In addition, we extended the investigation to other solvents, including mesitylene, dichloromethane and methanol. The results show that 3 exhibits comparable stability in dry mesitylene and dichloromethane, with half-lives of 53 minutes and 46 minutes, respectively (Fig. S17 and S18). In contrast, when dissolved in methanol, the recorded spectrum of the solution was identical to that of 2 in methanol (Fig. S19). Interestingly, the final product of 3 in different solvents all exhibited an absorption spectrum nearly identical to that of 2, confirming that 3 converts into 2 upon decay. This transformation is consistent with a protonation-coupled reduction pathway, analogous to the known phenoxyl radical to phenol conversion.^75–79^ To verify this, acetic acid was added to the toluene solution of 3, and the characteristic absorption band at 610 nm disappeared completely, leaving only the spectrum of 2 (Fig. 4c). In contrast, adding acetic acid to 2 or a reported phenoxyl radical derivative 2-OMe produced no change. These results demonstrate that acid accelerates the decay of 3. Cyclic voltammetry indicates that the reduction potential of 3 alone is insufficient to drive this process; however, protonation likely lowers the LUMO energy level, thereby facilitating reduction and promoting the conversion from 3 to 2 (Fig. 4d).

**Fig. 5:**
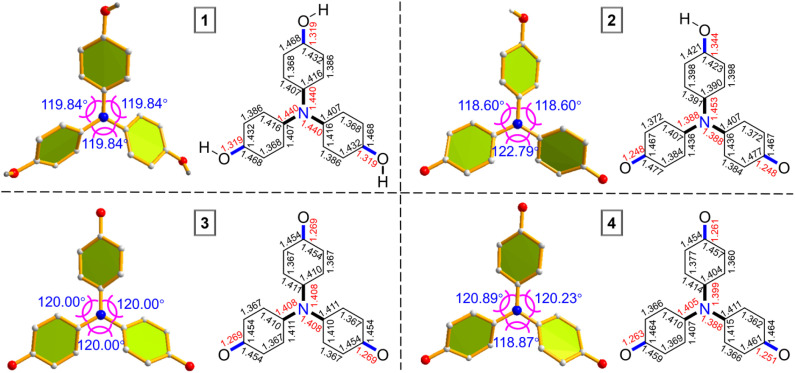
X-ray crystallographic analysis. X-ray structures and bond length (in Å) of the backbones of the single-crystal structures of 1, 2, 3 and 4. Solvent molecules and *n*-Bu_4_N^+^ anions of 4 were omitted for the sake of clarity. The three values around the nitrogen atom represented the angles between the three adjacent carbon-nitrogen bonds.

Single crystals of compounds 1–4 suitable for X-ray diffraction were obtained by slow diffusion of hexane into dichloromethane under inert conditions.^80^ Structural analysis revealed two distinct conformational types (Fig. 5). Compounds 1 and 3 exhibit *C*_3_ symmetry, with 3 showing a fully conjugated, nearly planar framework. In contrast, compounds 2 and 4 display *C*_2_ symmetry and a pronounced quinoidal character. These conformational differences arise from variations in electronic delocalization around the central nitrogen atom. In 1 and 3, the three bond angles (*θ*1–*θ*3) around N1 are nearly equivalent, consistent with their symmetrical geometries. For 2 and 4, the angles are markedly unequal (Table 1). The N1 atom in 1 lies 0.058 Å out of the C4–C7–C13 plane, indicative of a flattened tetrahedral geometry due to a localized nitrogen lone pair. By contrast, in 2–4, especially 3, N1 is essentially coplanar with adjacent atoms, reflecting delocalization of the lone pair over the extended π-system and favoring a fully planar structure. Bond length analysis further supports these findings (Table 1). 3 features highly delocalized phenoxyl units with uniform C–O bonds (1.269 Å), significantly shorter than the C–OH bonds in 1 and 2, yet slightly longer than a conventional C=O double bond. The C–N bonds (C4–N1, C7–N1, and C13–N1) in 3 and 4, as well as C7–N1 and C13–N1 in 2, are shorter than the single C–N bond in 1 (1.440 Å) (Fig. 5 and Table 1), highlighting enhanced conjugation. Bond length alternation (BLA) analysis of 3 reveals values intermediate between 1 (localized) and 2/4 (quinoidal), consistent with its fully delocalized electronic structure. To corroborate these interpretations, variable-temperature ^1^H NMR (VT-NMR) of 2 was performed. Upon heating, significant signal broadening in both aromatic and aliphatic regions (Fig. S12) was observed, confirming restricted C–N bond rotation and thus its partially conjugated, quinoidal nature.^71^

**Table 1:** Selected bond lengths, dihedral angles and BLA parameters of 1, 2, 3 and 4^a^

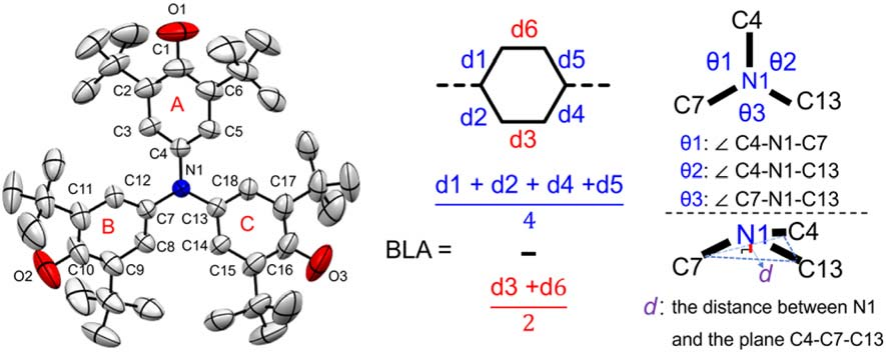				
	1	2	3	4
O1–C1 (Å)	1.319	1.344	1.269	1.261
O3–C16 (Å)	1.319	1.248	1.269	1.251
O2–C10 (Å)	1.319	1.248	1.269	1.263
C4–N1 (Å)	1.440	1.453	1.408	1.399
C7–N1 (Å)	1.440	1.388	1.408	1.405
C13–N1 (Å)	1.440	1.388	1.408	1.400
BLA(A) (Å)	0.0538	0.0085	0.0655	0.0638
BLA(B) (Å)	0.0538	0.0688	0.0655	0.0675
BLA(C) (Å)	0.0538	0.0688	0.0655	0.0738
*θ*1 (°)	119.84	118.60	120.00	120.89
*θ*2 (°)	119.84	118.60	120.00	120.23
*θ*3 (°)	119.84	122.19	120.00	118.87
*d* (Å)	0.058	0.000	0.000	0.008

a Bond lengths, dihedral angles and BLA parameters of 1, 2, 3 and 4 were determined from single crystals.

**Fig. 6:**
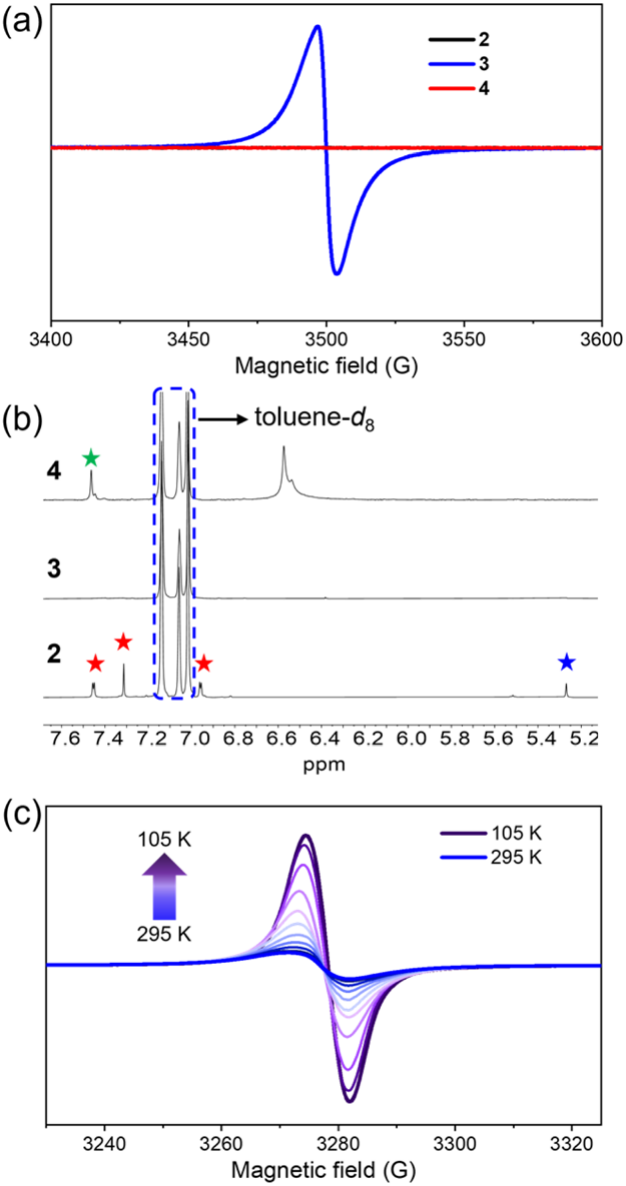
The investigation of the electronic structures of 2, 3 and 4. (a) EPR spectra of 2, 3 and 4 (∼4.4 × 10^−4^ M in toluene) at 298 K. Both 2 and 4 remain very weak EPR signals. Microwave frequency = 9.819 GHz, microwave power = 20.00 mW, modulation frequency = 100.0 kHz, modulation amplitude = 1.000 G, and sweep width = 200.0 G. (b) Partial ^1^H NMR spectra (300 MHz, toluene-*d*_8_, 298 K) of 2, 3 and 4. Note: the red stars represent the protons on the aromatic ring of 2; the blue star represents the proton on the hydroxyl group of 2; the green star represents the protons on the aromatic ring of 4; the signals in the blue box were derived from the protons of toluene-*d*_8_. (c) VT-EPR spectra of 3 (∼4.4 × 10^−4^ M in toluene). Microwave frequency = 9.174 GHz, microwave power = 20.00 mW, modulation frequency = 100.0 kHz, modulation amplitude = 1.000 G, and sweep width = 200.0 G.

The electronic structures of 2, 3 and 4 were further investigated using EPR and NMR spectroscopy. Both 2 and 4 were EPR silent (Fig. 6a), but showed sharp ^1^H NMR peaks (Fig. 6b), indicating that both 2 and 4 adopted closed-shell singlet ground states (Fig. 6a and b). This result was consistent with previous studies, indicating that such structures exhibited a distinct quinoidal character with a very small diradical contribution.^71,72^ In contrast, 3 showed a broad and intense EPR signal (Fig. 6a), while its ^1^H NMR spectrum at room temperature was completely broadened (Fig. 6b), indicating its paramagnetic behavior. The absence of well-resolved hyperfine splitting in the EPR spectra of 3 can be attributed to the delocalization of the unpaired electron over multiple aromatic rings leading to overlapping hyperfine features from several equivalent hydrogen atoms. The *g*-value of 3 is determined to be 2.0045 (Fig. S28). This *g*-value is characteristic of delocalized organic oxygen-centered radicals; the slightly stronger spin–orbit coupling of the oxygen atom results in a marginally higher *g*-value than that of typical carbon-centered radicals (≈2.0030). This observation supports that the unpaired electron is predominantly delocalized over the conjugated C–O π-system (phenoxyl moieties) rather than localized on a single heavy-atom center. Subsequently, the ground state of compound 3 was investigated by variable-temperature EPR (VT-EPR) spectroscopy in toluene over the temperature range of 105–295 K (Fig. 6c). Upon cooling, the EPR signal intensity of 3 gradually increased, a common behavior for paramagnetic monoradicals,^81^ attributable to the temperature-dependent Boltzmann distribution of spin populations. The corresponding *I versus* 1/*T* plot (where *I* represents the EPR signal intensity at each temperature and *T* represents the temperature) is shown in Fig. S30. The *I* value exhibited a linear correlation with 1/*T*, consistent with the Curie law for paramagnetic species. This behavior is characteristic of monoradicals with a doublet ground state, further confirming that compound 3 exists predominantly in a thermally stable open-shell state without significant thermal population of high spin states.

**Fig. 7:**
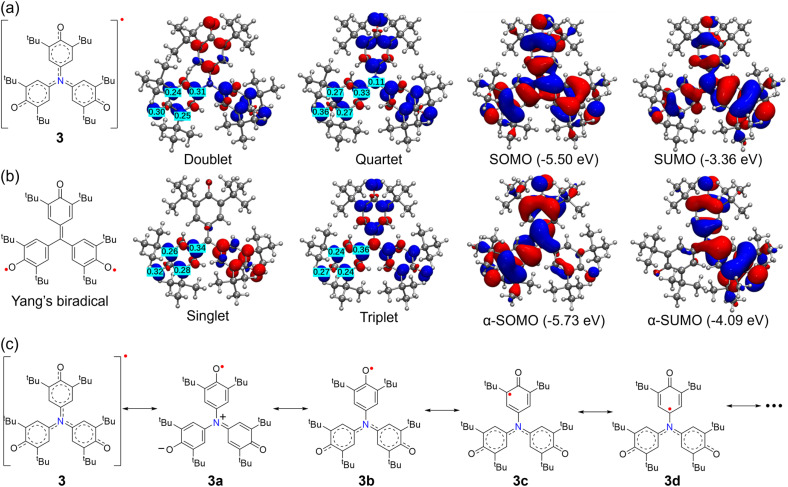
DFT calculations of 3 and Yang's biradical. Calculated spin density distributions and the frontier molecular orbitals (SOMO and SUMO) of (a) 3 and (b) Yang's biradical. Data calculated at the UB3LYP/6-31G(d) level of theory. (c) Selected canonical resonance structures of 3.

To gain deeper insight into the electronic structure of 3, we performed DFT calculations (UB3LYP/6-31G(d)) of its ground state, spin density, and frontier molecular orbitals, alongside a comparative study of Yang's biradical at the same level. The electronic structure was analyzed using Multiwfn.^82^ The results show that 3 adopts a doublet ground state with a large doublet-quartet energy gap (Δ*E*_D–Q_ ≈ −6.27 kcal mol^−1^) (Fig. S32), whereas Yang's biradical favors a triplet ground state (Δ*E*_S–T_ ≈ 5.42 kcal mol^−1^) (Fig. S35). The unpaired electron in 3 is extensively delocalized over the three phenoxyl units, mainly on the oxygen atoms and the *ortho*/*para* positions of the aromatic rings, with no spin density on the central nitrogen atom (Fig. 7a). This accounts for the absence of ^14^N hyperfine splitting in its EPR spectrum (Fig. 6a). In contrast, the singlet spin density of Yang's biradical is localized on just two phenoxyl moieties (Fig. 7a), though their triplet states exhibit similar distributions (Fig. 7b). The marked difference in ground-state multiplicity arises from the replacement of the central carbon in Yang's biradical with a π-conjugated sp^2^ nitrogen atom in 3. The nitrogen's lone pair engages in π-conjugation with adjacent phenyl rings, stabilizing the single unpaired electron and favoring a doublet state. In Yang's biradical, the carbon bridge lacks this conjugative stabilization, leading to two unpaired electrons and a triplet ground state, consistent with its trimethylenemethane analogue.^83^ Resonance analysis of 3 reveals contributions from ionic structures (*e.g.*, 3a) and delocalized quinoidal monoradical forms (3b–3d) (Fig. 7a). Frontier molecular orbital (FMO) analysis further supports these findings. 3 shows significant SOMO–SUMO overlaps with a large energy gap of 2.14 eV (Fig. 7a), matching the observed absorption maximum at 610 nm and confirming the high electronic stability of this delocalized open-shell species. Overall, nitrogen bridging in 3 fundamentally alters its electronic configuration compared to carbon-bridged Yang's biradical, conferring doublet character, enhanced planarity, and global π-delocalization.

## Conclusions

In this study, we have elucidated the structural and electronic features of a hitherto unknown 4,4′,4″-nitrilotriphenoxyl radical, compound 3, derived from Yang's biradical framework. Through a combination of single-crystal X-ray diffraction, variable-temperature EPR spectroscopy, and DFT calculations, 3 was unambiguously identified as a planar, *C*_3_-symmetric monoradical with a delocalized doublet ground state. 3 was a metastable species (half-life: 116 min), which would convert into hydroxyl precursor 2. Our study indicates the introduction of the sp^2^-hybridized nitrogen bridge plays a pivotal role in modulating the spin distribution and ground-state multiplicity, distinguishing 3 fundamentally from Yang's biradical, which adopts a triplet ground state. Beyond the synthesis and characterization of 3, the interconversion among hydroxyl precursor 2 and its deprotonated anionic form 4, and 3 showcases a redox-responsive system spanning closed- and open-shell species, enabling dynamic modulation of the electronic structure. These findings not only enrich the understanding of nitrogen-bridged polyphenoxyl radicals, but also establish a rational framework for designing new redox-active open-shell materials with tailored spin and electronic properties.

## Author contributions

X. S., H.-B. Y., Q.-Y. H. and B. H. conceived the project, analyzed the data, and wrote the manuscript. Q.-Y. H. and B. H. performed most of the experiments. X.-L. Z. and Y. N. conducted single crystal analyses. C. W. conducted MS analysis. All authors discussed the results and commented on the manuscript.

## Conflicts of interest

The authors declare no competing financial interest.

## Supplementary Material

SC-017-D5SC06789H-s001

SC-017-D5SC06789H-s002

## Data Availability

Supplementary information (SI): The Supporting Information is available free of charge on the ACS Publications website at. experimental details, additional characterizations and figures including synthetic route, ^1^H and ^13^C NMR spectra, 2D NMR spectra, MS spectra, and single crystal analysis (PDF). See DOI: https://doi.org/10.1039/d5sc06789h. CCDC 2454706 (1), 2454730 (2), 2454731 (3) and 2454733 (4) contain the supplementary crystallographic data for this paper.^84*a*–*d*^
