# Supplementary material for: A 4,4′,4″-nitrilotriphenoxyl radical derived from Yang's biradical
Source: Chem Sci. 2025 Nov 27;17(3):1583–91. doi: 10.1039/d5sc06789h (PMC12683543; doi:10.1039/d5sc06789h)
Supplement: SC-017-D5SC06789H-s001 [file SC-017-D5SC06789H-s001.pdf]

Supplementary Information (SI) for Chemical Science.
This journal is © The Royal Society of Chemistry 2025

Supplementary Information

A 4,4',4''-Nitrilotriphenoxyl Radical Derived from Yang's Biradical

Qiong-Yan Hong,[a] Bin Huang,[a] Yanfei Niu,[a] Cuihong Wang,[a] Xiao-Li Zhao,[a] Hai-Bo Yang[a] and Xueliang Shi[a]*

[a] Q.-Y. Hong, Dr. B. Huang, Y. Niu, Prof. C. Wang, Prof. X.-L. Zhao, Prof. H.-B. Yang, Prof. X. Shi
State Key Laboratory of Petroleum Molecular & Process Engineering, Shanghai Key Laboratory of Green Chemistry and Chemical Processes, School of Chemistry and Molecular Engineering, East China Normal University, Shanghai 200062, China

*Corresponding author. E-mail: xlshi@chem.ecnu.edu.cn

Table of Contents

1. Materials, instruments and methods 2
2. Synthesis and characterization 2
3. X-ray crystallographic analysis 15
4. Cyclic voltammogram and differential pulse voltammetry of **2-OMe**, **2** and **3** 19
5. EPR measurements 22
6. Theoretical calculation 24
7. Reference 31
8. Cartesian coordinates 32

1. Materials, instruments and methods

All reagents and starting materials were obtained from commercial suppliers and used without further purification. The amine-bridged derivative of Galvinoxyl radical **2-OMe** was synthesized according to the literature.[1] All air-sensitive reactions were carried out under inert N_2 atmosphere. The ^{1}H NMR, ^{13}C NMR spectra were recorded in solution of $CDCl_3$, C_6D_6 and toluene-d_8 on Bruker 300 MHz, Bruker 400 MHz, and Bruker 500 MHz spectrometer. 2D NMR (COSY, DOSY) was measured in solution of $CDCl_3$ and toluene-d_8 on Bruker 400 MHz spectrometer. Coupling constants (J) are denoted in Hz and chemical shifts (δ) are denoted in ppm. Multiplicities are denoted as follows: s = singlet, d = doublet, b = broaden and m = multiplet. All Fourier-transform infrared (FT-IR) spectra were collected on a TENSOR ll FT-IR spectrometer. Mass spectra were obtained with a Bruker micro TOF-Q II mass spectrometer (Bruker Daltonics Corp., USA) and a Waters Synapt G2 mass spectrometer in the electrospray ionization (ESI) mode, dichloromethane as solvent. The single crystals of this work were measured on Rigaku XtaLAB PRO MM003-DS dual system with Cu Kα radiation (λ = 1.54184 Å). Cyclic voltammetry was recorded on a Bio-Logic SAS SP-150 spectrometer in anhydrous DCM or in dry solution (DCM/toluene = 1/4, v/v) containing Bu_4NPF_6 (0.1 M) as supporting electrolyte at a scan rate of 20 mV/s at room temperature. The CV cell has a glassy carbon electrode, a Pt wire counter electrode, and an Ag/Ag^+ reference electrode. The potential was externally calibrated against the ferrocene/ferrocenium (Fc/Fc^+) couple. The highest occupied molecular orbital (HOMO) and lowest unoccupied molecular orbital (LUMO) energy levels were calculated based on the equations: $E_{HOMO/LUMO} = -(4.80 + E^{ox}_{onset}/E^{red}_{onset})$ eV. EPR spectra for radicals were obtained on Bruker EMX instrument EMXPLUS-10/12.

2. Synthesis and characterization

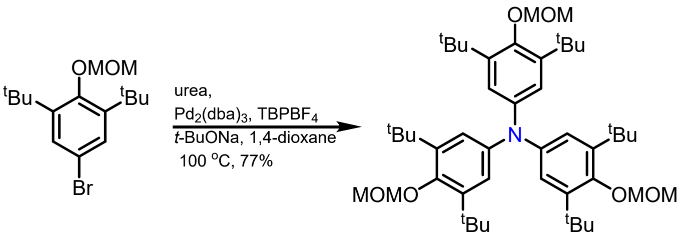

5 6

Compound 6: Under the protection of nitrogen, 4-bromo-2,6-di-*tert*-butylphenol methoxymethyl ether (1.92 g, 5.83 mmol), urea (0.10 g, 1.66 mmol), $Pd_2(dba)_3$ (305 mg, 0.333 mmol), $(tert\text{-}Bu)_3P•HBF_4$ (194 mg, 0.666 mmol), and potassium tert-butoxide (2.24 g, 23.31 mmol) were added into a dry Schlenk tube. Then 20 mL of dry 1,4-dioxane was added to disperse the powder. After that, the mixture was stirred at 100 °C for 24 h. After cooling to

room temperature, undissolved solid was filtered. The filtrate was concentrated under reduced pressure to obtain the crude product, which was purified give pure compound **6** as a white solid (0.98 g, 77%). ^{1}H NMR (500 MHz, Chloroform-*d*) δ 6.92 (s, 6H), 4.89 (s, 6H), 3.62 (s, 9H), 1.34 (s, 54H). ^{13}C NMR (126 MHz, Chloroform-*d*) δ 149.05, 144.35, 142.66, 121.64, 100.45, 57.35, 35.81, 31.99. HR-ESI-TOF MS: m/z = 761.5575 (calculated for $C_{48}H_{75}NO_6$: 761.5594).

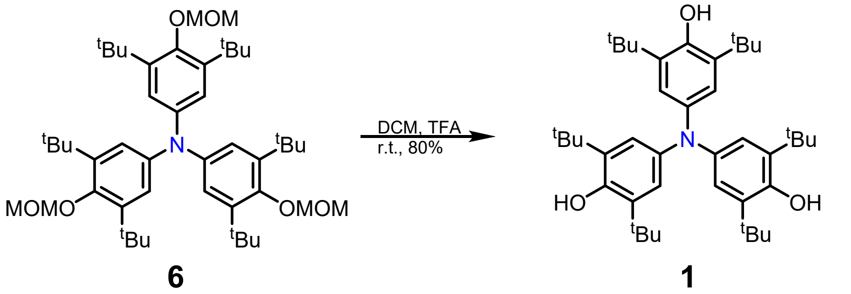

Compound 1: **6** (100 mg, 0.13 mmol) was dissolved with CH_2Cl_2 (5 ml), and TFA (3 ml) was added to the solution. After stirring for 1 h at room temperature, the reaction mixture was dried in vacuo and dissolved in CH_2Cl_2 and then washed with water. The organic layer was evaporated, and the crude product was chromatographed on silica gel (hexane/DCM = 2/1 as eluent) to afford **1** (66 mg, 80%) as gray solid. ^{1}H NMR (500 MHz, Chloroform-*d*) δ 6.90 (s, 6H), 4.83 (s, 3H), 1.35 (s, 54H). ^{13}C NMR (126 MHz, Chloroform-*d*) δ 148.55, 140.10, 136.26, 120.04, 34.51, 30.38. HR-ESI-TOF MS: m/z = 629.4792 (calculated for $C_{42}H_{63}NO_3$: 629.4808).

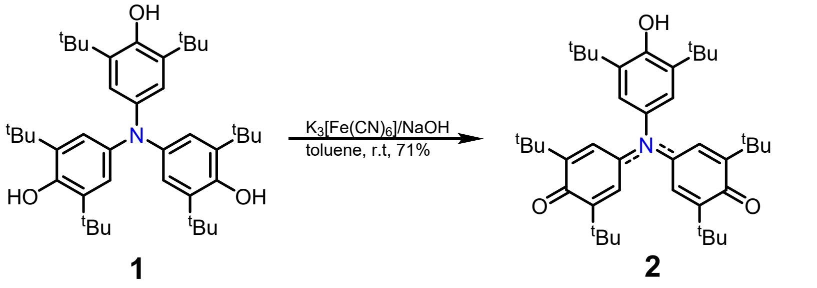

2: In a two-necked flask, **1** (0.050 g 0.08 mmol) was placed and toluene (10 ml) was added under argon atmosphere. To the flask, a solution of $K_3[Fe(CN)_6]$ (0.527 g 1.6 mmol) and NaOH (77 mg 1.92 mmol) in water (5 ml) was added and the reaction mixture was stirred for 1 h at room temperature. The organic layer was separated and washed with water. The organic layer was dried over Na_2SO4 and the solvent was removed by rotary evaporation. The crude product was chromatographed on silica gel (hexane/ethyl acetate = 10/1 as eluent) to afford **2** (0.035 g, 71 %) as deep blue solid. ^{1}H NMR (400 MHz, toluene-d_8) δ 7.45 (d, J = 2.9 Hz, 2H), 7.31 (s, 2H), 6.96 (d, J = 2.9 Hz, 2H), 5.27 (s, 1H), 1.53 (s, 18H), 1.42 (s, 18H), 1.21 (s, 18H).

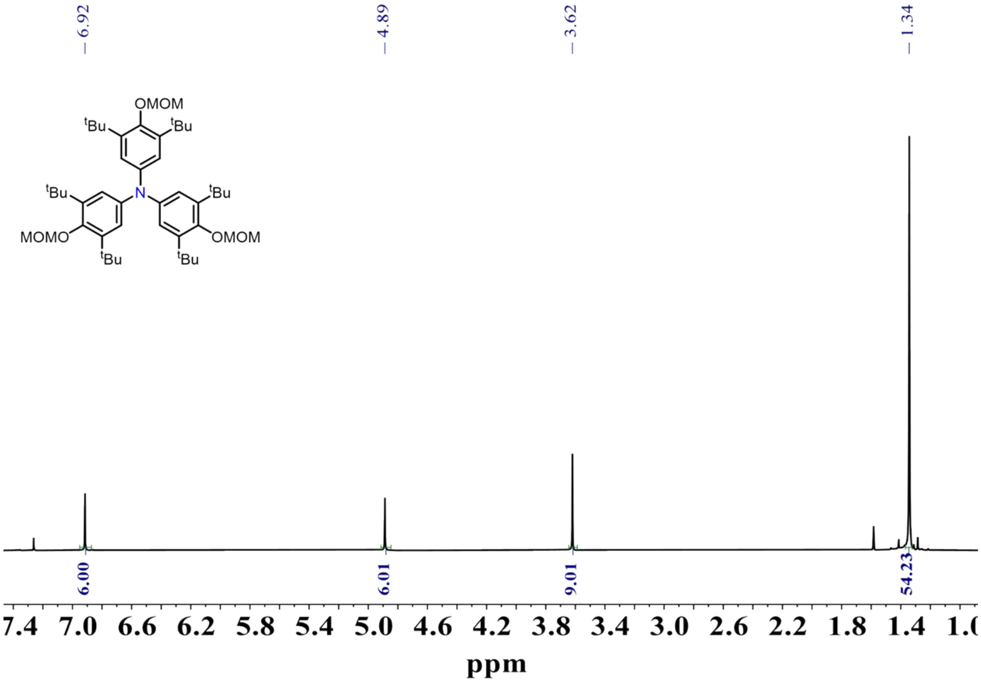

Fig. S1 ^{1}H NMR spectrum (500 MHz, $CDCl_3$, 298 K) of compound **6**.

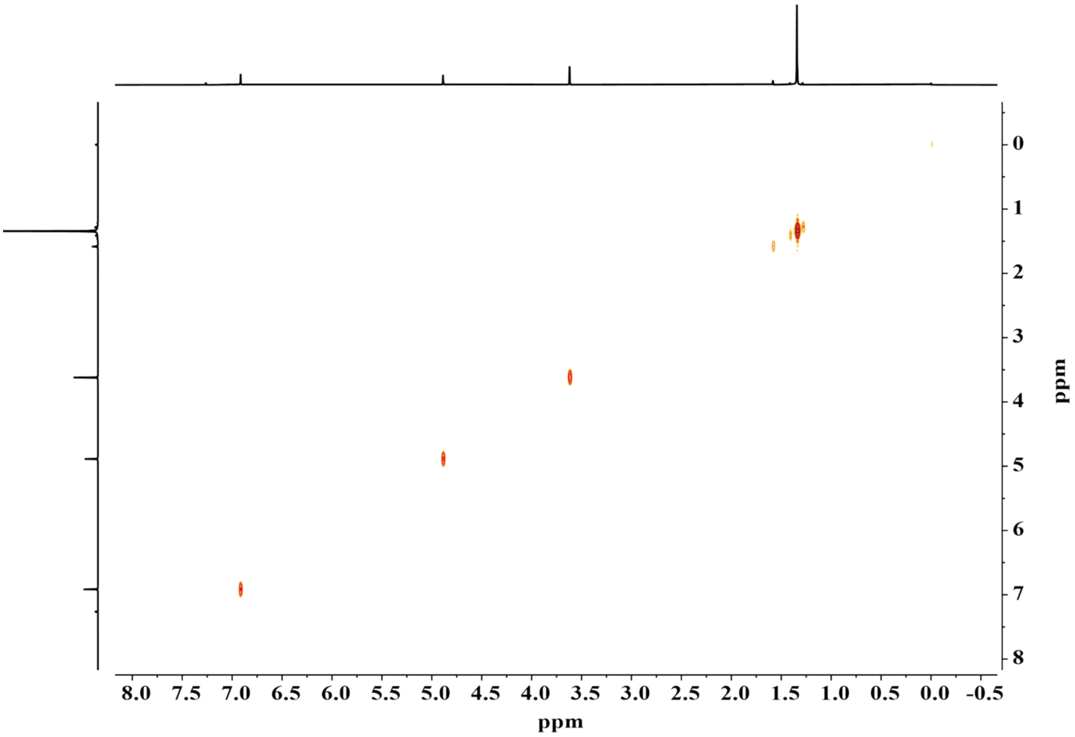

Fig. S2 2D COSY NMR spectrum (500 MHz, $CDCl_3$, 298 K) of compound **6**.

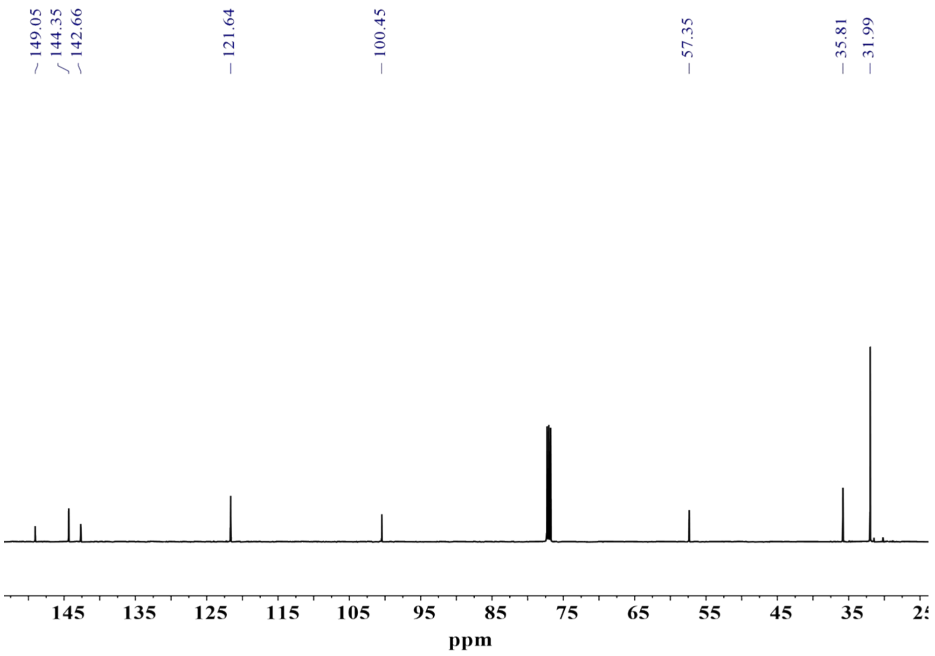

Fig. S3 ^{13}C NMR spectrum (126 MHz, $CDCl_3$, 298 K) of the compound **6**.

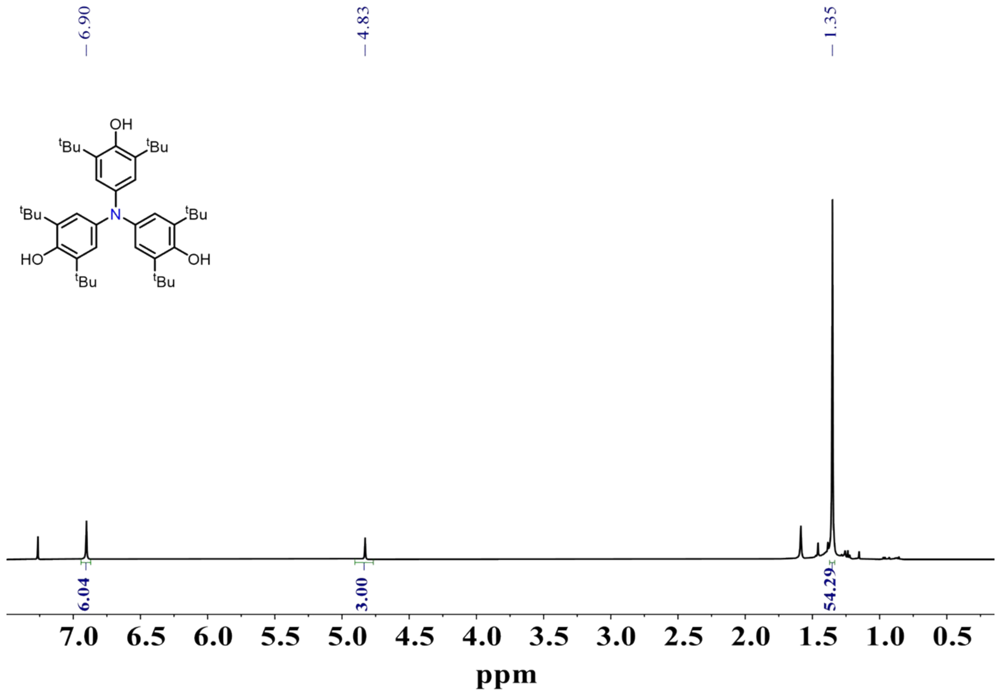

Fig. S4 ^{1}H NMR spectrum (500 MHz, $CDCl_3$, 298 K) of compound **1**.

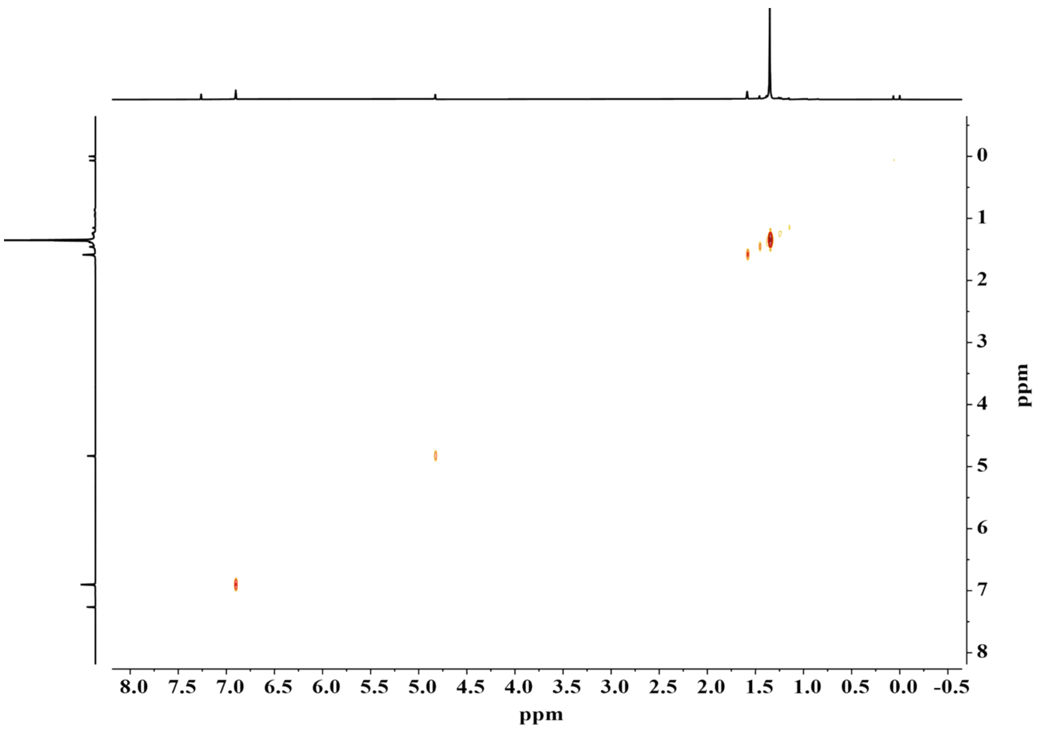

Fig. S5 2D COSY NMR spectrum (500 MHz, $CDCl_3$, 298 K) of compound **1**.

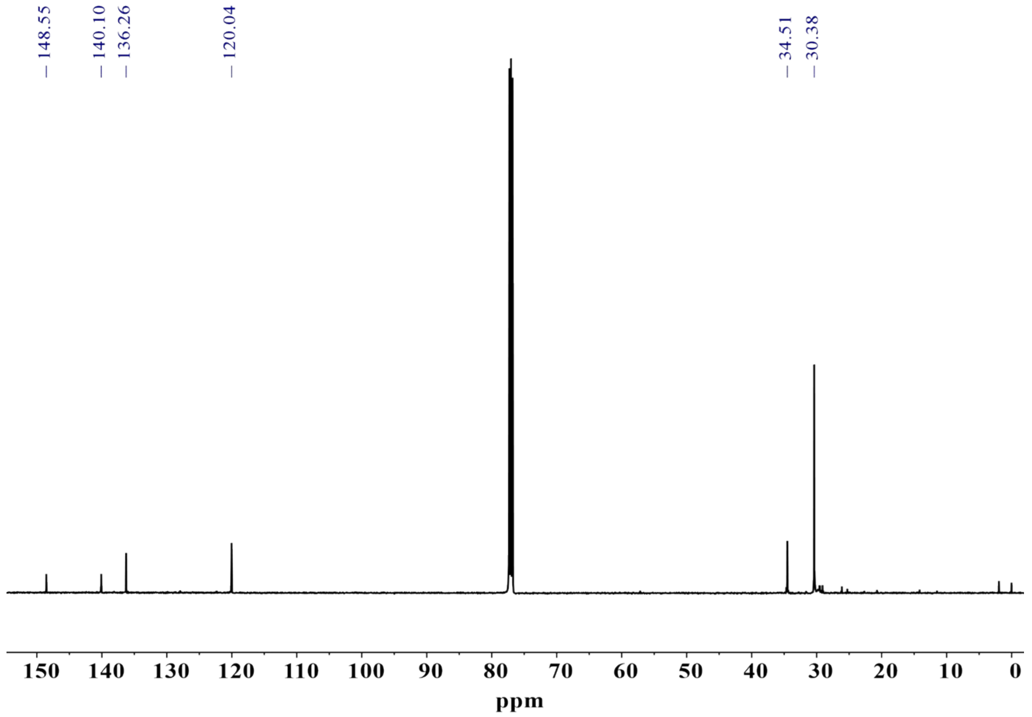

Fig. S6 ^{13}C NMR spectrum (126 MHz, $CDCl_3$, 298 K) of compound **1**.

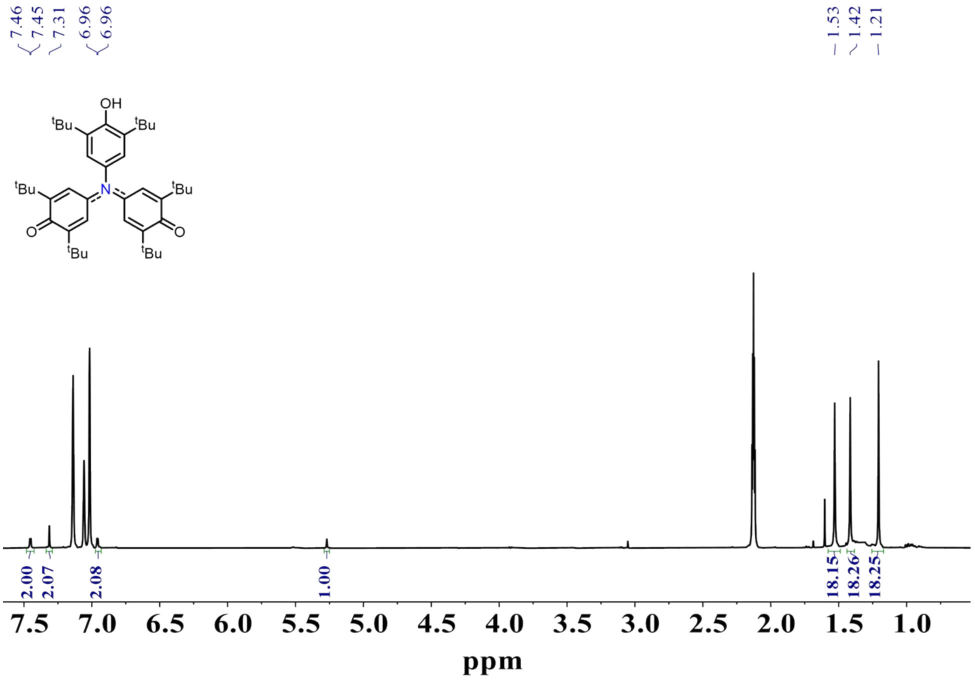

Fig. S7 ^{1}H NMR spectrum (500 MHz, toluene-d_8, 298 K) of compound **2**.

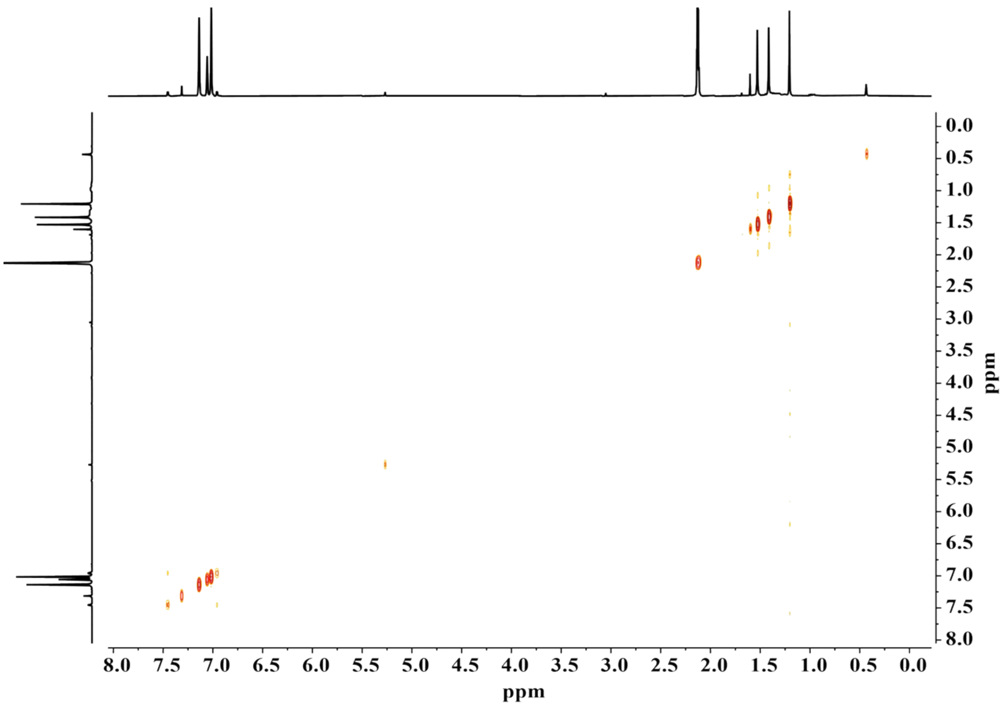

Fig. S8 2D COSY NMR spectrum (500 MHz, toluene-d_8, 298 K) of compound **2**.

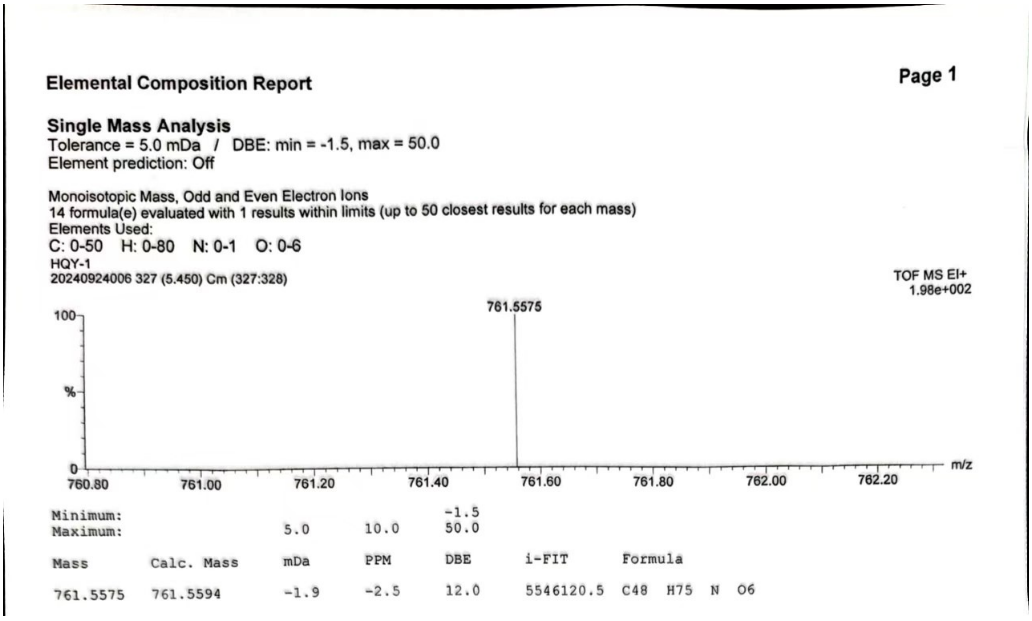

Elemental Composition Report — **Page 1**

Single Mass Analysis
Tolerance = 5.0 mDa / DBE: min = -1.5, max = 50.0
Element prediction: Off

Monoisotopic Mass, Odd and Even Electron Ions
14 formula(e) evaluated with 1 results within limits (up to 50 closest results for each mass)
Elements Used:
C: 0-50 H: 0-80 N: 0-1 O: 0-6
HQY-1
20240924006 327 (5.450) Cm (327:328)

Minimum:				-1.5		
Maximum:		5.0	10.0	50.0		
Mass	Calc. Mass	mDa	PPM	DBE	i-FIT	Formula
761.5575	761.5594	-1.9	-2.5	12.0	5546120.5	C48 H75 N O6

Fig. S9 HR-ESI-MS spectrum of compound **6**.

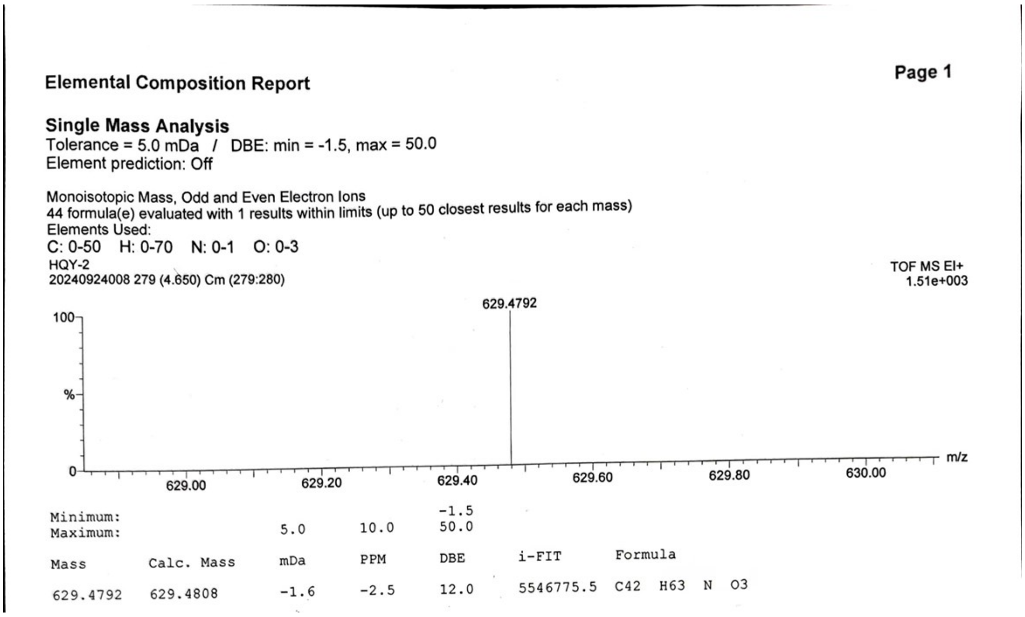

Elemental Composition Report — **Page 1**

Single Mass Analysis
Tolerance = 5.0 mDa / DBE: min = -1.5, max = 50.0
Element prediction: Off

Monoisotopic Mass, Odd and Even Electron Ions
44 formula(e) evaluated with 1 results within limits (up to 50 closest results for each mass)
Elements Used:
C: 0-50 H: 0-70 N: 0-1 O: 0-3
HQY-2
20240924008 279 (4.650) Cm (279:280)

Minimum:				-1.5		
Maximum:		5.0	10.0	50.0		
Mass	Calc. Mass	mDa	PPM	DBE	i-FIT	Formula
629.4792	629.4808	-1.6	-2.5	12.0	5546775.5	C42 H63 N O3

Fig. S10 HR-ESI-MS spectrum of compound **1**.

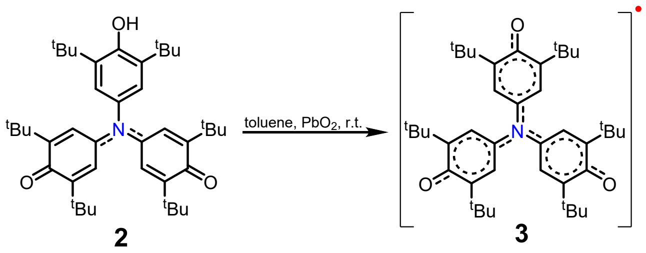

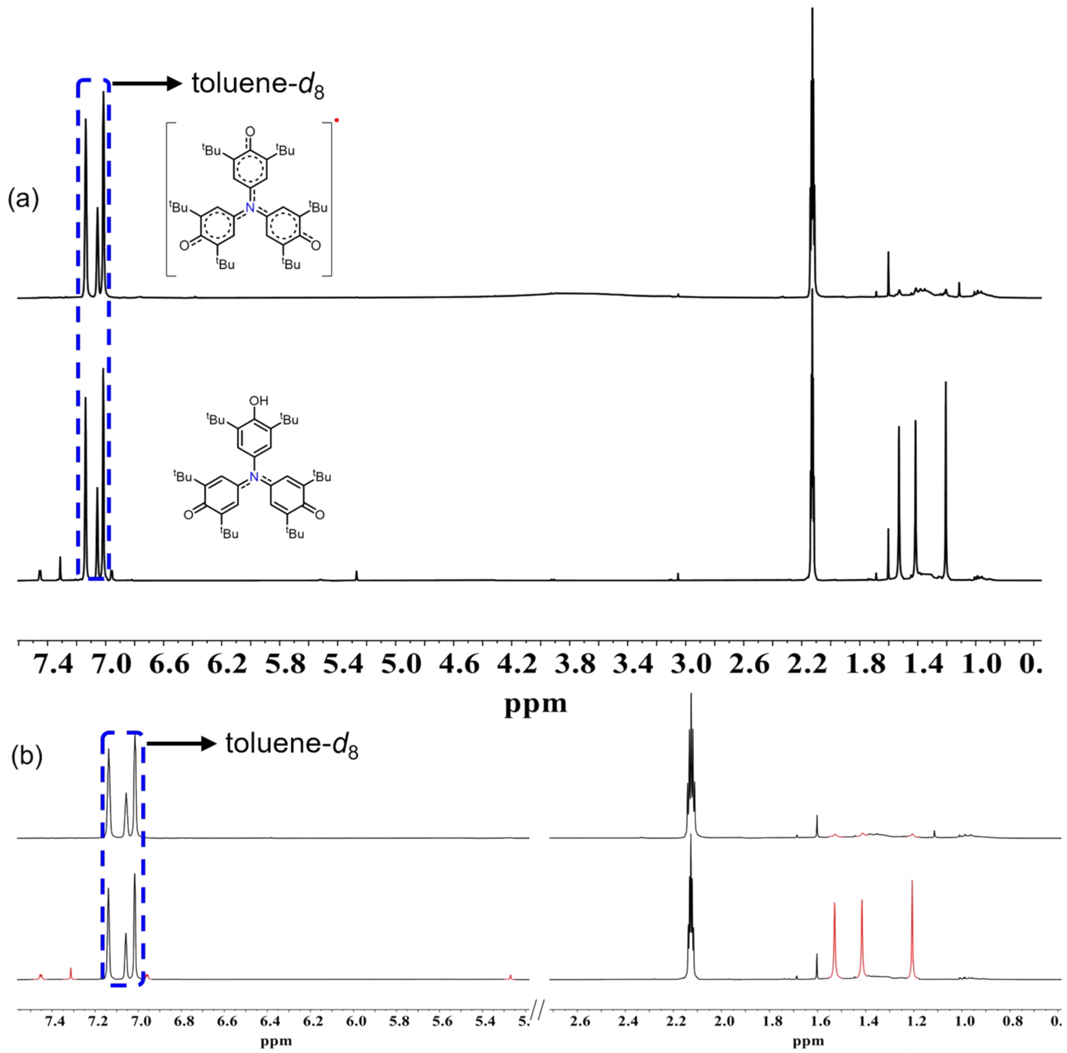

Fig. S11 Toluene solution of **2** was treated with excessive PbO_2. (a) ^1H NMR spectra (300 MHz, toluene-d_8, 298 K) of **2** and **3**. (b) Expanded ^1H NMR spectra (300 MHz, toluene-d_8, 298 K) of **2** and **3**.

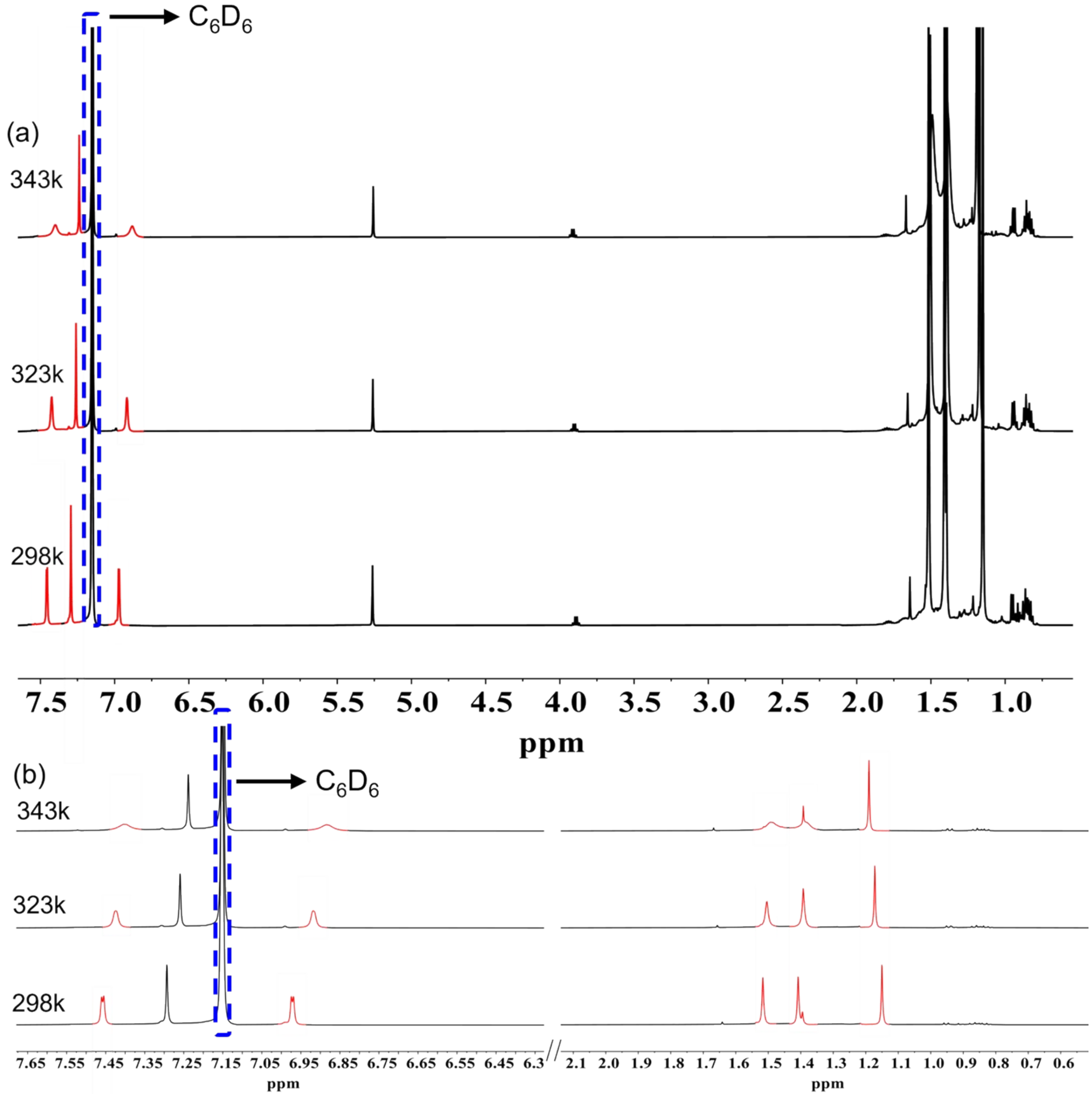

Fig. S12 (a) Variable-temperature ^{1}H NMR spectra (300 MHz, toluene-d_8) of **2**. (b) Expanded variable-temperature ^{1}H NMR spectra (300 MHz, toluene-d_8) of **2**.

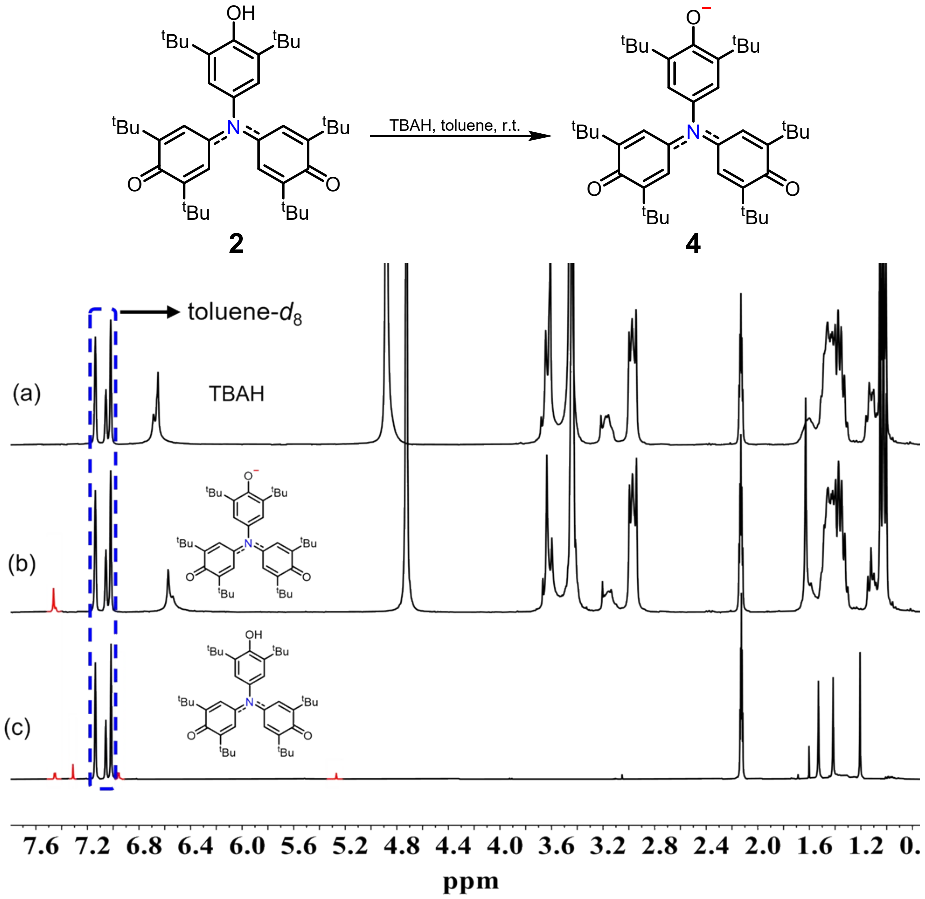

Fig. S13 Toluene solution of **2** was treated with excessive TBAH. (a) ^{1}H NMR spectrum (300 MHz, toluene-d_8, 298 K) of **TBAH**. (b) ^{1}H NMR spectrum (300 MHz, toluene-d_8, 298 K) of **4**. (c) ^{1}H NMR spectrum (300 MHz, toluene-d_8, 298 K) of **2**.

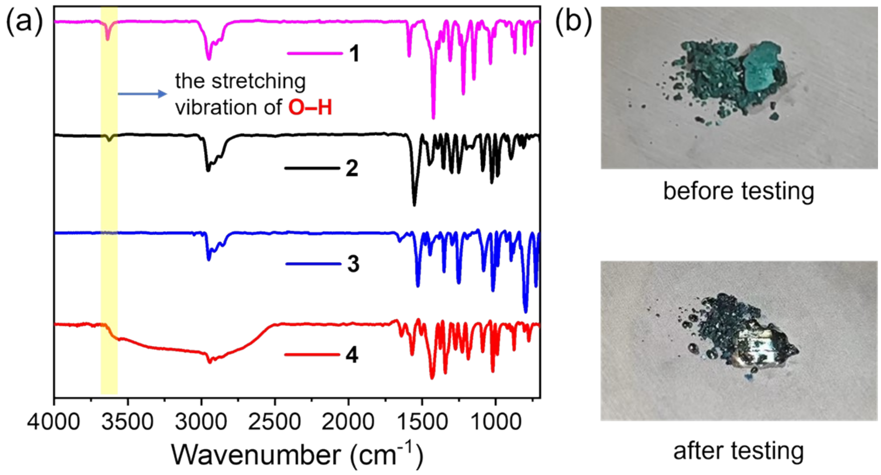

Fig. S14 (a) FT-IR spectra of **1**, **2**, **3** and **4** in solid state. (b) Photos of **4** before and after FT-IR testing.

Note: the FT-IR spectrum of **4** shows a broad absorption band in the 2500–3700 cm^{-1} region, which can be attributed to water absorption, as this compound is highly hygroscopic (Fig. S14b).

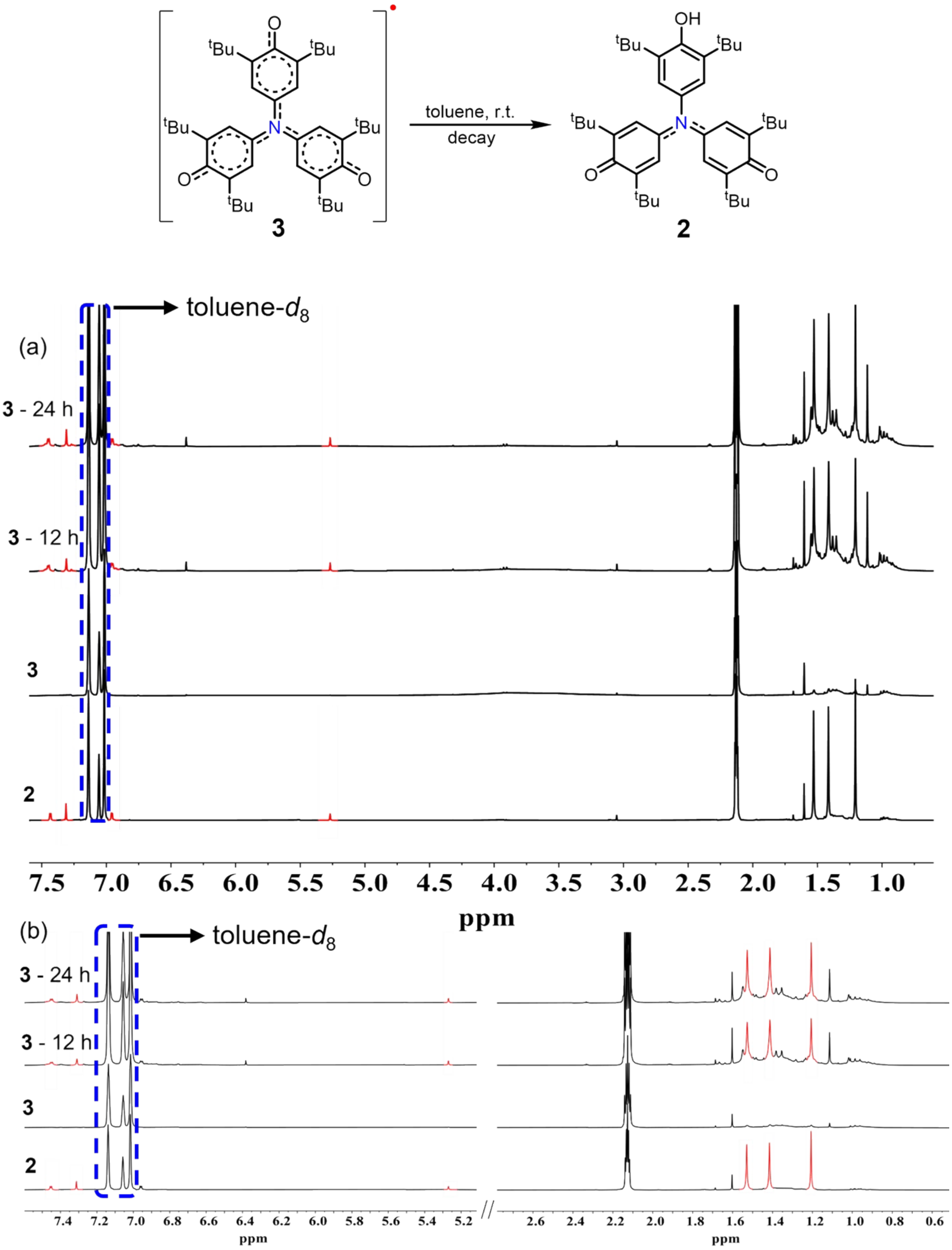

Fig. S15 In toluene at room temperature, **3** undergoes gradual reduction to **2**. (a) Time dependent ^{1}H NMR spectra (300 MHz, toluene-d_8) of **3**, and ^{1}H NMR spectrum (300 MHz, toluene-d_8) of **2**. (b) Expanded time dependent ^{1}H NMR spectra (300 MHz, toluene-d_8) of **3**, and ^{1}H NMR spectrum (300 MHz, toluene-d_8) of **2**.

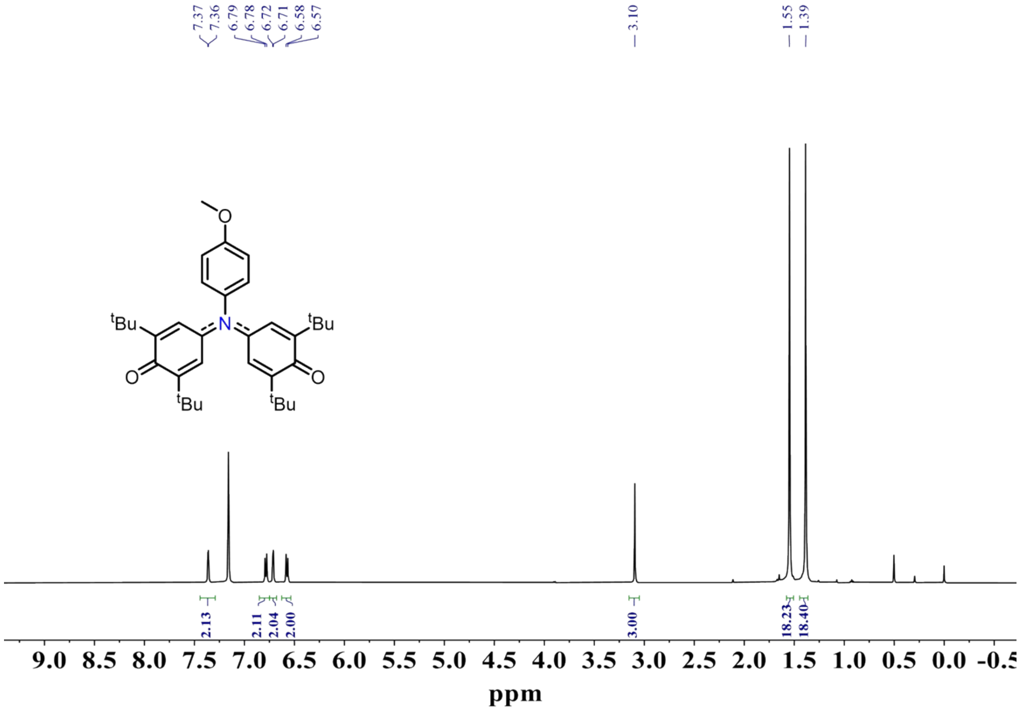

Fig. S16 ^{1}H NMR spectrum (500 MHz, C_6D_6, 298 K) of compound **2-OMe**. The spectrum was consistent with the literature.[1]

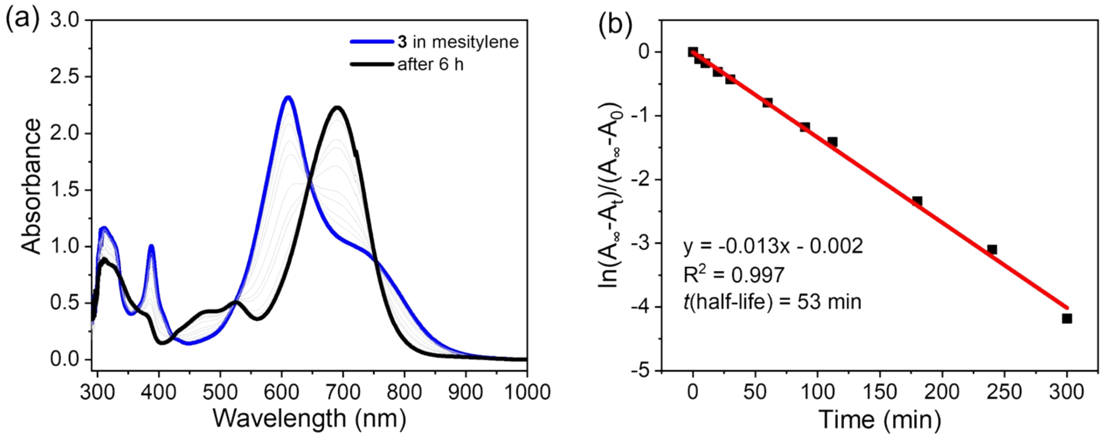

Fig. S17 (a) Change of UV-vis-NIR absorption spectra of **3** (~4.4×10^{-4} in mesitylene) over time. (b) Stability plot of **3** in mesitylene.

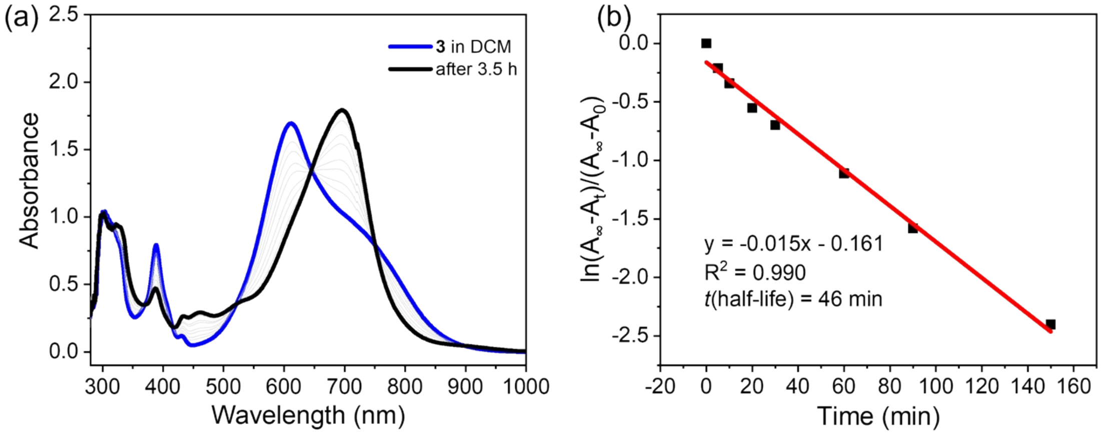

Fig. S18 (a) Change of UV-vis-NIR absorption spectra of **3** (~4.4×10^{-4} in DCM) over time. (b) Stability plot of **3** in DCM.

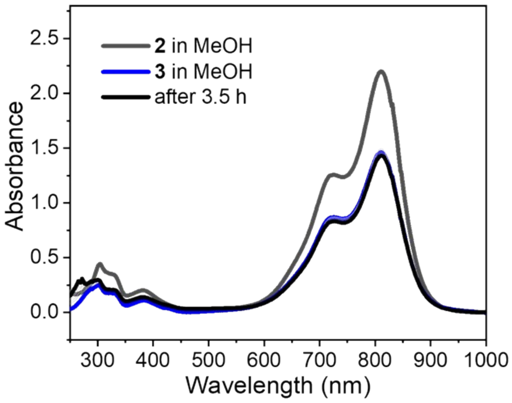

Fig. S19 Change of UV-vis-NIR absorption spectra of **3** (~4.4×10−4 in MeOH) over time.

3. X-ray crystallographic analysis

The single crystals of **1**, **2**, **3** and **4** were obtained by the slow evaporation of a mixed solution (dichloromethane and methanol). Data collections were performed on a Rigaku XtaLAB PRO MM003-DS dual system with Cu Kα radiation (λ = 1.54184 Å) at 173 K.

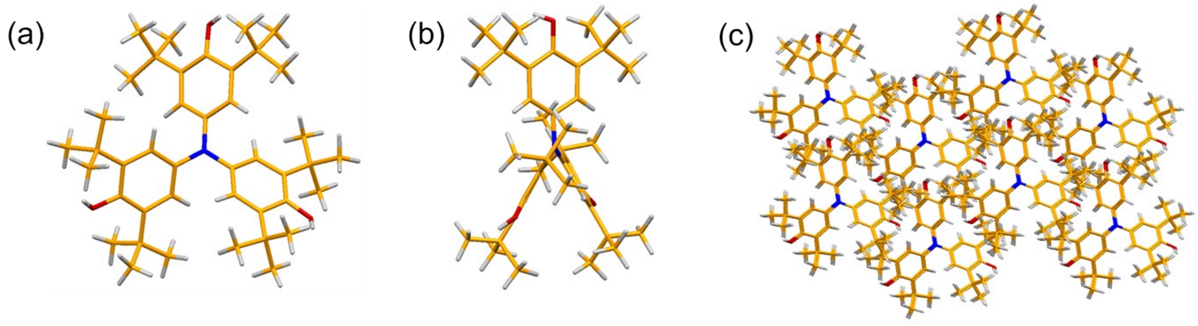

Fig. S20 Single crystal structure and molecular packing of **1**. Solvent molecules were omitted for the sake of clarity.

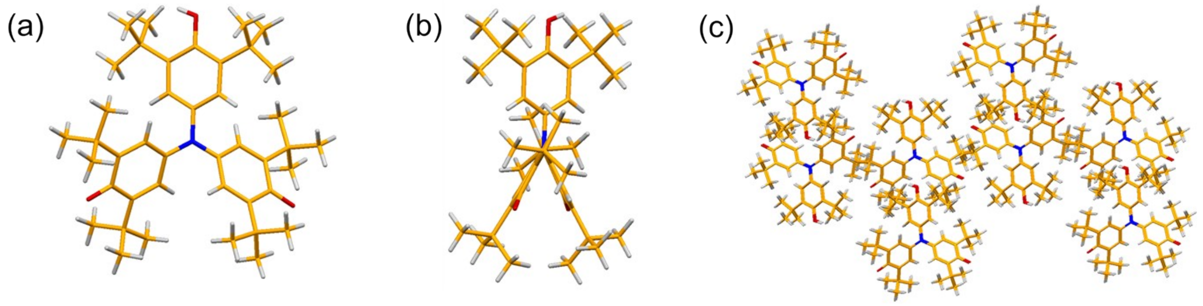

Fig. S21 Single crystal structure and molecular packing of **2**. Solvent molecules were omitted for the sake of clarity.

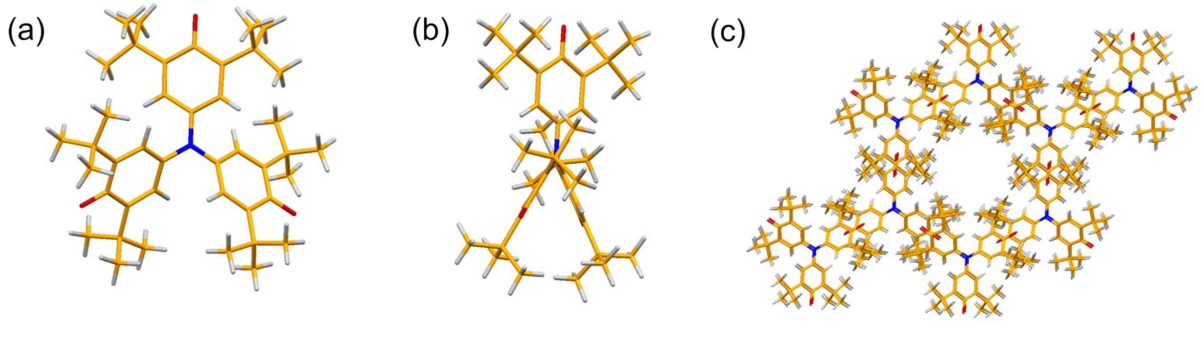

Fig. S22 Single crystal structure and molecular packing of **3**.

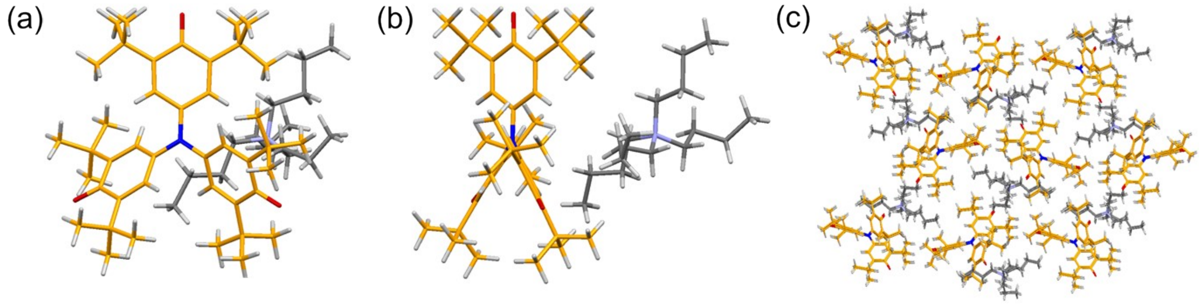

Fig. S23 Single crystal structure and molecular packing of **4**.

Table S1. The X-ray single crystal data of **1**, and **2**.

**Compound**	**1**	**2**
Empirical formula	$C_{42}H_{63}NO_3$	$C_{42}H_{61}NO_3$
Formula weight	629.93	627.95
Temperature / K	173.00(10)	172.99(10)
Crystal system	trigonal	monoclinic
Space group	*R*3	*C*2/*c*
a / Å	15.8786(7)	11.4459(11)
b / Å	15.8786(7)	17.3442(12)
c / Å	17.1045(9)	22.2295(17)
α / °	90	90
β / °	90	100.430(8)
γ / °	120	90
Volume / $Å^3$	3734.8(4)	4340.1(6)
Z	3	8
$\rho_{calc}g / cm^3$	0.848	1.102
μ / mm^{-1}	0.394	0.510
F (000)	1038.0	1576.0
Crystal size / mm^3	0.32 × 0.26 × 0.22	0.22 × 0.16 × 0.08
Radiation	Cu Kα (λ = 1.54184)	Cu Kα (λ = 1.54184)
2Θ range for data collection / °	8.25 to 133.872	8.088 to 134.152
Index ranges	$-18 \le h \le 17$, $-18 \le k \le 18$, $-20 \le l \le 20$	$-9 \le h \le 13$, $-20 \le k \le 20$, $-25 \le l \le 26$
Reflections collected	12362	23071
Independent reflections	2901 $R_{int} = 0.0425$, $R_{sigma} = 0.0336$	3877 $R_{int} = 0.1282$, $R_{sigma} = 0.0918$
Data / restraints / parameters	2901 / 366 / 285	3877 / 136 / 284
Goodness-of-fit on F^2	1.021	1.198
Final R indexes [I>=2σ (I)]	$R_1 = 0.0732$, $wR_2 = 0.2145$	$R_1 = 0.1398$, $wR_2 = 0.3199$
Final R indexes	$R_1 = 0.0873$, $wR_2 = 0.2338$	$R_1 = 0.1867$, $wR_2 = 0.3542$
Largest diff. peak / hole / e $Å^{-3}$	0.31 / -0.14	1.19 / -0.30
CCDC	2454706	2454730

Table S2. The X-ray single crystal data of **3** and **4**.

**Compound**	**3**	**4**
Empirical formula	$C_{42}H_{60}NO_3$	$C_{58}H_{96}N_2O_3$
Formula weight	626.91	626.91
Temperature / K	173.00(10)	172.99(10)
Crystal system	trigonal	monoclinic
Space group	*P*-31c	$P2_1$/c
a / Å	15.3640(13)	10.2641(4)
b / Å	15.3640(13)	26.5484(9)
c / Å	11.5469(12)	22.5350(9)
α / °	90	90
β / °	90	97.544(4)
γ / °	120	90
Volume / Å^3	2360.5(5)	6087.5(4)
Z	2	4
ρ_{calc}g / cm^3	0.882	0.949
μ / mm^{-1}	0.415	0.429
F (000)	686.0	1928.0
Crystal size / mm^3	0.26 × 0.22 × 0.18	0.38 × 0.26 × 0.22
Radiation	Cu Kα (λ = 1.54184)	Cu Kα (λ = 1.54184)
2Θ range for data collection / °	6.644 to 133.996	7.748 to 134.15
Index ranges	-18 ≤ h ≤ 17, -18 ≤ k ≤ 18, -13 ≤ l ≤ 11	-7 ≤ h ≤ 12, -31 ≤ k ≤ 31, -26 ≤ l ≤ 26
Reflections collected	26025	68425
Independent reflections	1410 R_{int} = 0.1321, R_{sigma} = 0.0531	10828 R_{int} = 0.0821, R_{sigma} = 0.0608
Data / restraints / parameters	1410 / 0 / 75	10828 / 51 / 590
Goodness-of-fit on F^2	1.087	1.081
Final R indexes [I>=2σ (I)]	R_1 = 0.0955, wR_2 = 0.2938	R_1 = 0.0857, wR_2 = 0.2411
Final R indexes	R_1 = 0.1134, wR_2 = 0.3109	R_1 = 0.1213, wR_2 = 0.2691
Largest diff. peak / hole / e Å^{-3}	0.27 / -0.22	0.70 / -0.40
CCDC	2454731	2454733

4. Cyclic voltammogram and differential pulse voltammetry of 2-OMe, 2 and 3

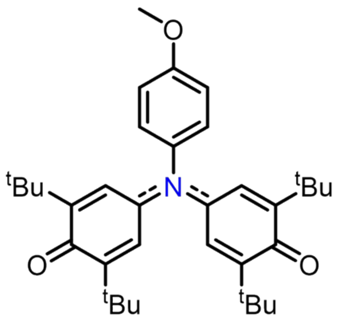

2-OMe

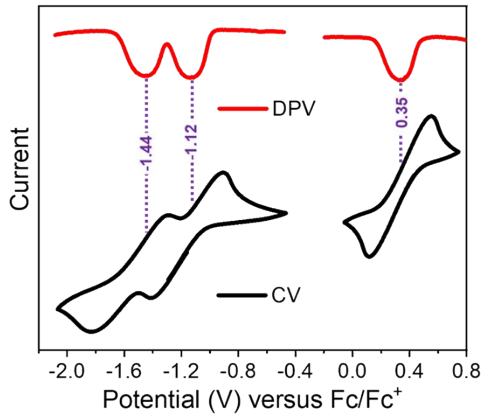

Fig. S24 Cyclic voltammogram (CV) and differential pulse voltammogram (DPV) curves of **2-OMe**[1] measured in dry solution (DCM/toluene = 1/4, v/v) with 0.1 M *n*-Bu_4NPF_6 as the supporting electrolyte, Ag/AgCl as the reference electrode, a Pt wire as the counter electrode and a scan rate at 20 mV/s.

According to the literature,[1] compound **2-OMe** exhibits one reversible reduction and one reversible oxidation process. Under identical experimental conditions, we re-evaluated its electrochemical behavior. The results revealed that **2-OMe** in fact undergoes two reversible reduction processes and one reversible oxidation process. Notably, the first reduction potential and the oxidation potential are consistent with those previously reported.[1]

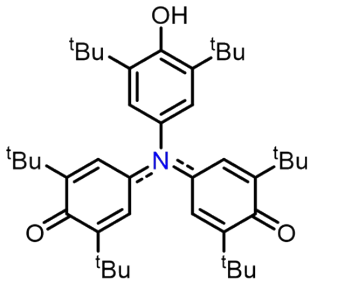

2

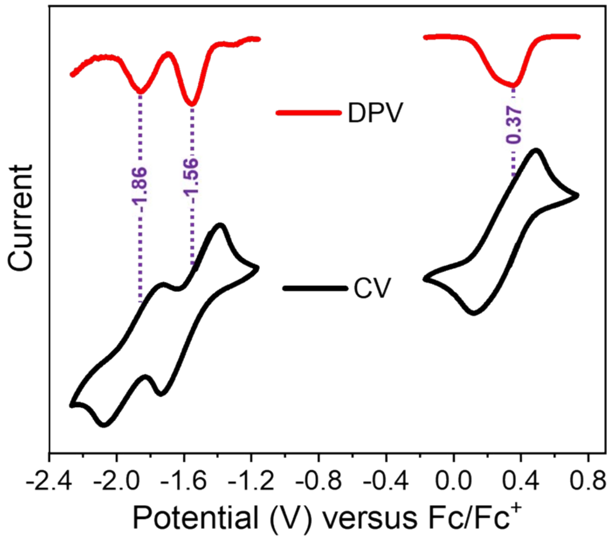

Fig. S25 Cyclic voltammogram (CV) and differential pulse voltammogram (DPV) curves of **2** measured in dry solution (DCM/toluene = 1/4, v/v) with 0.1 M n-Bu_4NPF_6 as the supporting electrolyte, Ag/AgCl as the reference electrode, a Pt wire as the counter electrode and a scan rate at 20 mV/s.

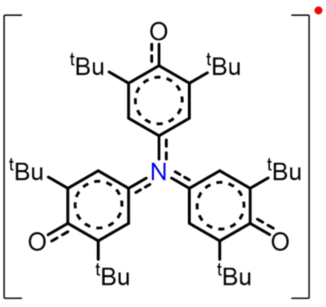

3

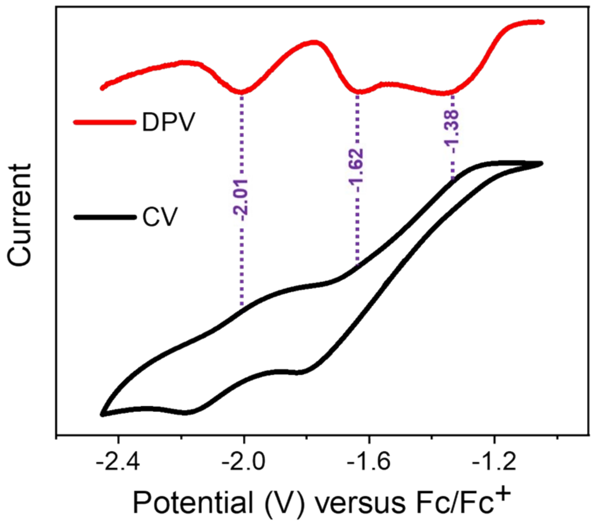

Fig. S26 Cyclic voltammogram (CV) and differential pulse voltammogram (DPV) curves of **3** measured in dry solution (DCM/toluene = 1/4, v/v) with 0.1 M n-Bu_4NPF_6 as the supporting electrolyte, Ag/AgCl as the reference electrode, a Pt wire as the counter electrode and a scan rate at 20 mV/s.

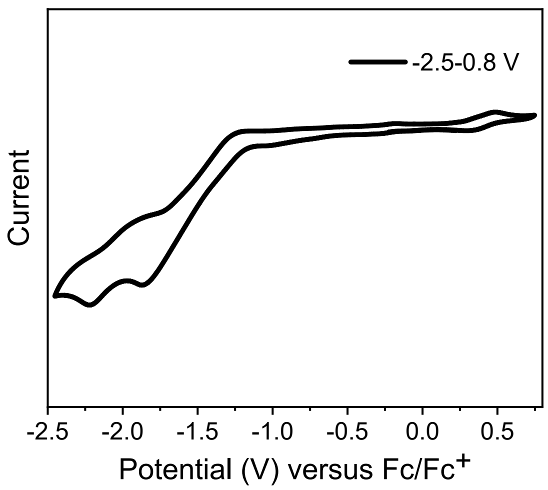

Fig. S27 Cyclic voltammogram and differential pulse voltammetry of **3** in dry solution (DCM/toluene = 1/4, v/v) with 0.1 M Bu_4NPF_6 as the supporting electrolyte, Ag/AgCl as the reference electrode, a Pt wire as the counter electrode and a scan rate at 20 mV/s.

5. EPR measurements

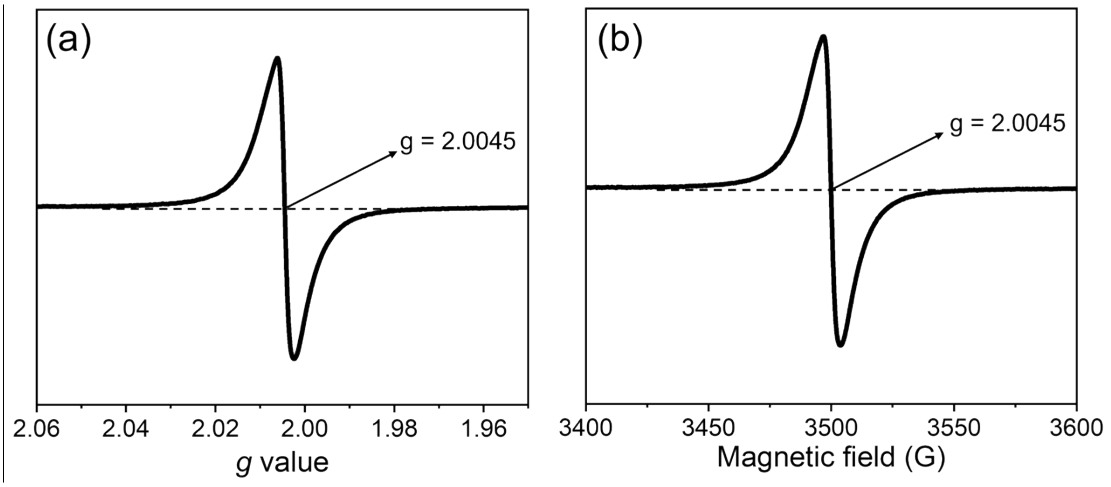

Fig. S28 EPR spectra of **3** in toluene. Microwave frequency = 9.819 GHz, microwave power = 20.00 mW, modulation frequency = 100.0 kHz, modulation amplitude = 1.000 G, and sweep width = 200.0 G, *g*-value = 2.0045. (a) The plot is presented with the *g*-value as the x-axis. (b) The plot is presented with the magnetic field as the x-axis.

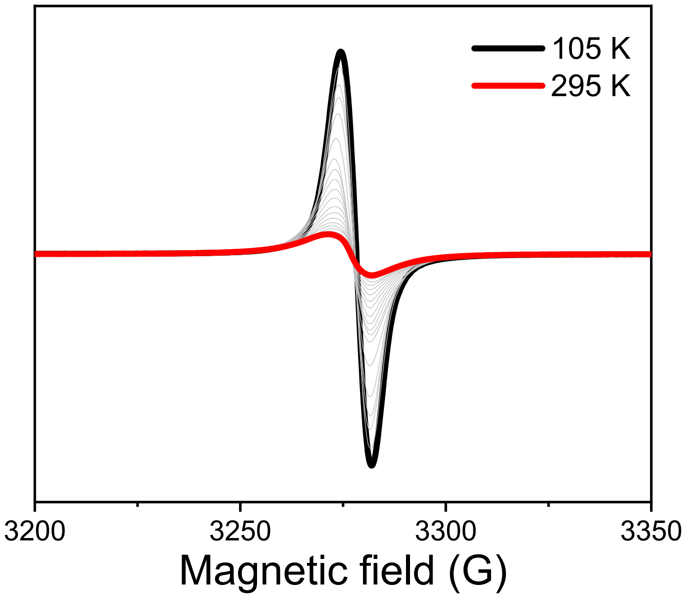

Fig. S29 VT-EPR spectra of **3** (~4.4×10^{-4} M in toluene).

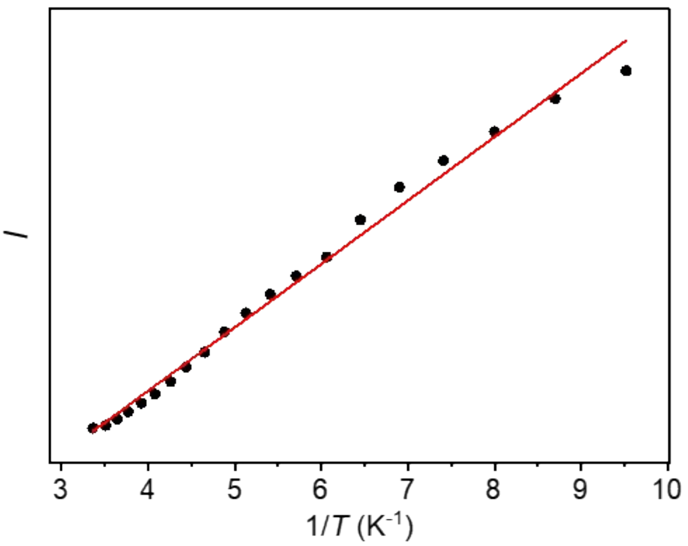

Fig. S30 Plot of integrated EPR intensity (*I*) of **3** against reciprocal temperature (1/*T*).

6. Theoretical calculation

Theoretical calculations were performed with the Gaussian16 program suite.[2] All molecules geometry optimizations were performed by using B3LYP exchange-correlation functional in conjunction with 6-31G(d) basis set.[3,4] Initial guess broken-symmetry (open-shell singlet) wavefunction was found with Guess=Mix, Nosymm and Stable=Opt keywords, then the Guess=Read keyword was used to optimize the broken-symmetry state geometry structure at the UB3LYP/6-31G(d) level. Frequency calculations were conducted to ensure that these structures were indeed local minima. Transition state structures were verified by frequency calculations and only one imaginary frequency was found in the transition state. Closed-shell wavefunction has an RHF→UHF instability and open-shell wavefunction is stable under the perturbations considered in stability test of wavefunction.[5-7] Single point energy was performed at the level of UB3LYP/6-31G(d).[8] Time-dependent density functional theory (TD-DFT) calculations were performed at the level of UB3LYP/6-31G(d). Spin population calculations based on becke method divide atomic space. Electronic structure analyses were performed with the Multiwfn 3.8 (dev) code. The isosurface maps of spin density and were rendered by means of Visual Molecular Dynamics (VMD 1.9.3) software[9] based on the files exported from Multiwfn.

The singlet-triplet energy gap $\Delta E_{S\text{-}T}$ was calculated as:

$\Delta E_{S\text{-}T} = E_{BS} - E_{T}$

where the E_{BS} and E_{T} were the energy of local minima structure in broken-symmetry state (= E_{S}: open-shell singlet) and triplet state, respectively.

The doublet- quartet energy gap $\Delta E_{D\text{-}Q}$ was calculated as:

$\Delta E_{D\text{-}Q} = E_{D} - E_{Q}$

where the E_{D} and E_{Q} were the energy of local minima structure in doublet state and quartet state, respectively.

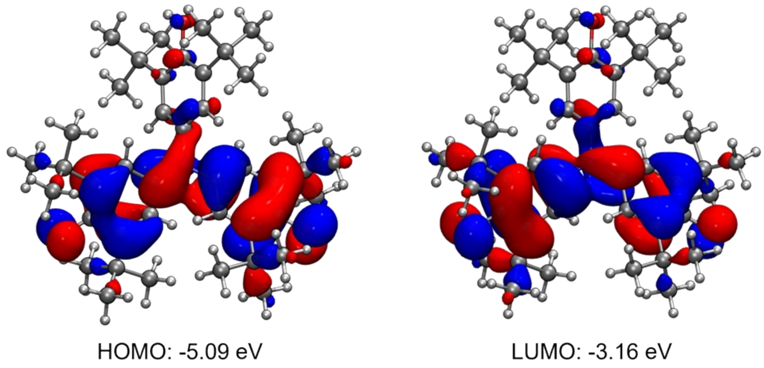

Fig. S31 Molecular orbitals of **2** in closed-shell state. The calculations were carried out at the level of B3LYP/6-31G(d).

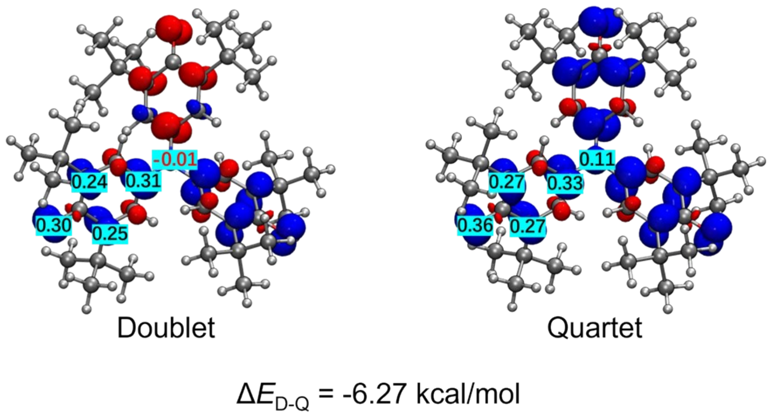

Fig. S32 Calculated spin density and spin population of **3** in doublet state (D) and quartet state (Q). The calculations were carried out at the level of UB3LYP/6-31G(d).

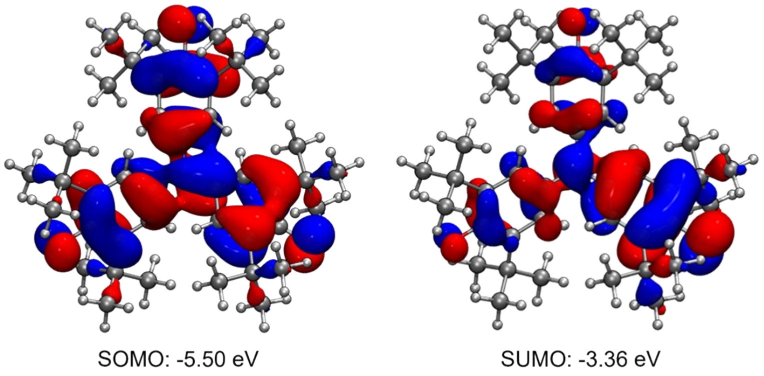

Fig. S33 Molecular orbitals of **3** in open-shell doublet state. The calculations were carried out at the level of UB3LYP/6-31G(d).

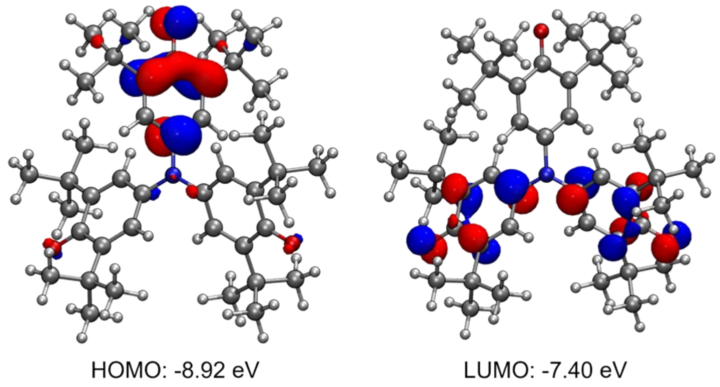

Fig. S34 Molecular orbitals of **4** in closed-shell state. The calculations were carried out at the level of B3LYP/6-31G(d).

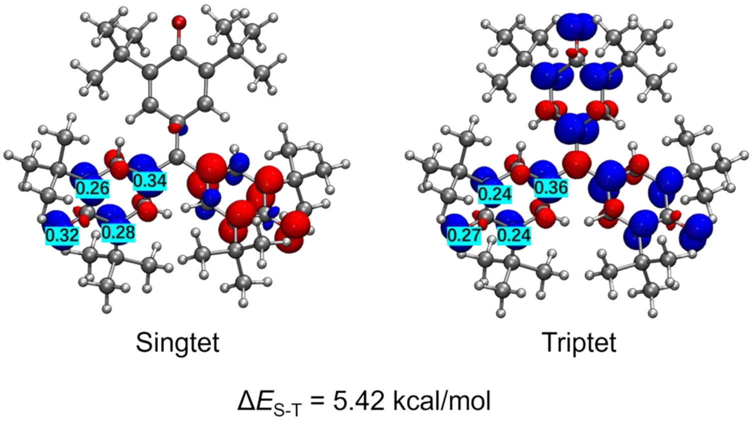

Fig. S35 Calculated spin density and spin population of Yang's biradical in open-shell singlet state (S) and triplet state (T). The calculations were carried out at the level of UB3LYP/6-31G(d).

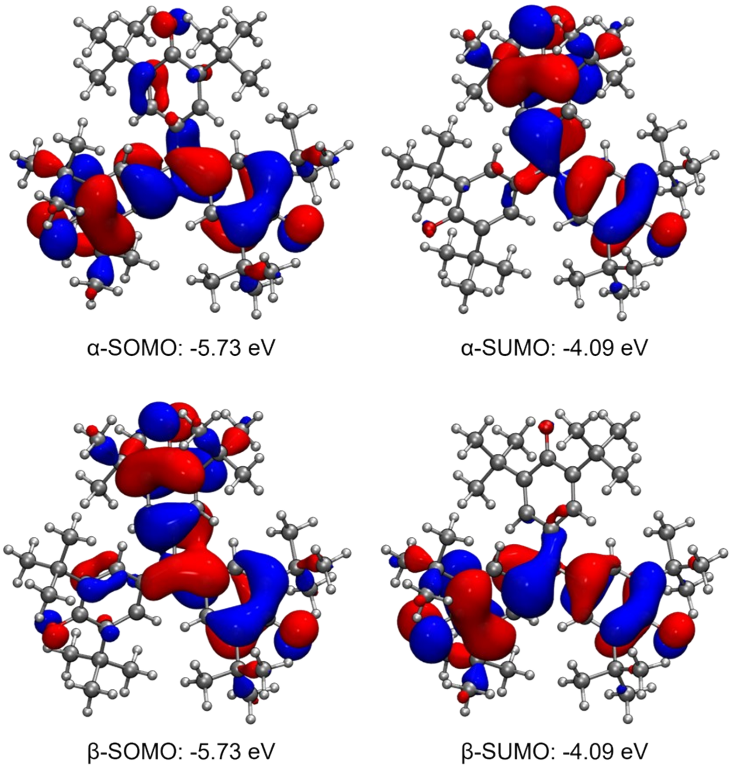

Fig. S36 Molecular orbitals of Yang's biradical in open-shell singlet state. The calculations were carried out at the level of UB3LYP/6-31G(d).

Table S3. HOMO–LUMO (or SOMO-SUMO for **3**) energy levels estimated experimentally and those obtained from DFT calculations at the level of UB3LYP/6-31G(d)

Compound	HOMO-LUMO energy level (eV)	
	experiment	calculation
**2**	1.56[a]	1.93
**3**	1.79[b]	2.14
**4**	1.32[b]	1.52

[a]The data were obtained from the cyclic voltammetry experiment.

[b]The data were obtained from the optical spectra. $\Delta E_{SOMO-SUMO} = E_{opt} = \frac{1240}{\lambda_{onset}}$

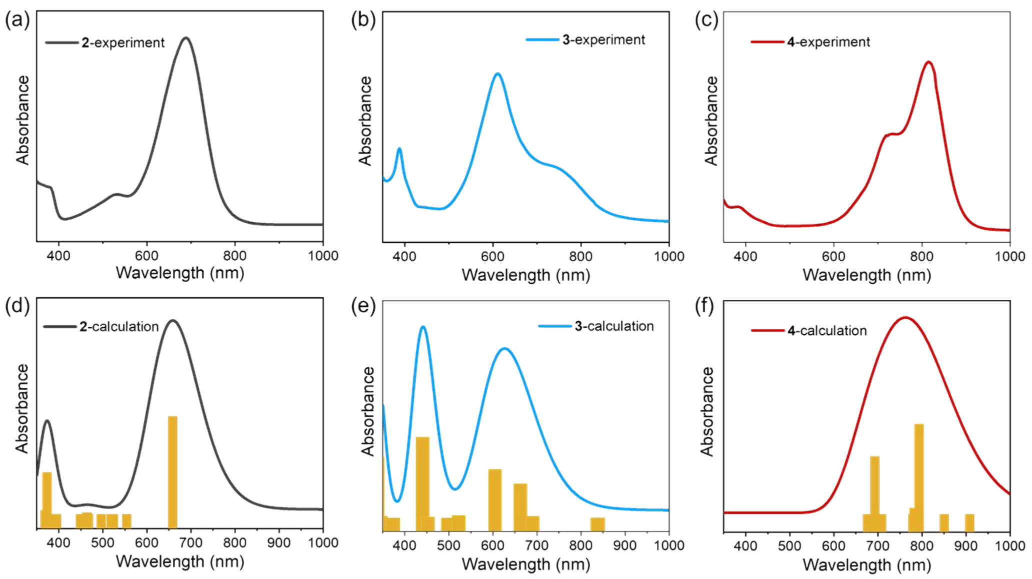

Fig. S37 Experimental absorption spectra of (a) **2**, (b) **3** and (c) **4** (~4.4×10^{-4} M in toluene). Calculated absorption spectra of (d) **2**, (e) **3** and (f) **4** at the level of uwb97xd/def2tzvp.

Table S4. Calculated absorption wavelengths (λ), oscillator strengths (f), and wavefunctions of **2** at the uwb97xd/def2tzvp level

State	Energy [eV]	λ [nm]	*f*	Orbitals(coefficient)
S1	-1.19	-1045	0	HOMO(A)->LUMO(A) (49%), HOMO(B)->LUMO(B) (49%)
S2	1.88	658	0.6778	HOMO(A)->LUMO(A) (51%), HOMO(B)->LUMO(B) (51%)
S3	2.24	555	0	H-1(A)->LUMO(A) (31%), H-1(B)->LUMO(B) (31%)
S4	2.37	524	0	H-2(A)->LUMO(A) (37%), H-2(B)->LUMO(B) (37%)
S5	2.39	519	0	H-3(A)->LUMO(A) (28%), H-3(B)->LUMO(B) (28%)
S6	2.50	496	0	H-4(A)->LUMO(A) (40%), H-4(B)->LUMO(B) (40%)
S7	2.65	467	0.0045	H-3(A)->LUMO(A) (15%), H-1(A)->LUMO(A) (25%), H-3(B)->LUMO(B) (15%), H-1(B)->LUMO(B) (25%)
S8	2.67	464	0.0123	H-2(A)->LUMO(A) (39%), H-2(B)->LUMO(B) (39%)
S9	2.76	449	0	H-5(A)->LUMO(A) (38%), H-5(B)->LUMO(B) (38%)
S10	3.14	395	0.002	H-4(A)->LUMO(A) (44%), H-4(B)->LUMO(B) (44%)
S11	3.30	376	0	H-7(A)->LUMO(A) (16%), H-7(B)->LUMO(B) (16%)
S12	3.32	373	0.2908	H-3(A)->LUMO(A) (27%), H-1(A)->LUMO(A) (14%), H-3(B)->LUMO(B) (27%), H-1(B)->LUMO(B) (14%)
S13	3.36	369	0.0272	H-5(A)->LUMO(A) (42%), H-5(B)->LUMO(B) (42%)

S14	3.67	338	0	HOMO(A)->L+1(A) (16%), HOMO(A)->L+3(A) (22%), HOMO(B)->L+1(B) (16%), HOMO(B)->L+3(B) (22%)
S15	3.83	324	0	H-6(A)->LUMO(A) (14%), H-6(B)->LUMO(B) (14%)

Table S5. Calculated absorption wavelengths (λ), oscillator strengths (f), and wavefunctions of **3** at the uwb97xd/def2tzvp level

State	Energy [eV]	λ [nm]	f	Orbitals(coefficient)
S1	1.48	839	0.0013	H-3(B)->LUMO(B) (57%), H-2(B)->L+1(B) (31%)
S2	1.48	837	0	H-3(B)->L+1(B) (31%), H-2(B)->LUMO(B) (58%)
S3	1.80	690	0.0065	H-2(A)->LUMO(A) (84%)
S4	1.87	663	0.1262	HOMO(B)->LUMO(B) (87%)
S5	2.05	605	0.1794	HOMO(A)->LUMO(A) (81%)
S6	2.38	522	0.0093	H-7(B)->L+1(B) (14%), H-5(B)->LUMO(B) (72%)
S7	2.50	498	0.0001	H-7(B)->LUMO(B) (41%), H-5(B)->L+1(B) (26%), H-4(B)->LUMO(B) (22%)
S8	2.74	453	0.0036	H-5(A)->LUMO(A) (54%), H-3(A)->LUMO(A) (26%)
S9	2.82	441	0.2987	HOMO(B)->L+1(B) (76%)
S10	3.32	374	0.0004	H-1(A)->LUMO(A) (23%), HOMO(A)->L+2(A) (10%), H-6(B)->LUMO(B) (37%)
S11	3.59	346	0.0065	H-8(A)->LUMO(A) (13%), H-6(B)->L+1(B) (13%), HOMO(B)->L+3(B) (14%)
S12	3.67	338	0.2238	H-1(A)->LUMO(A) (62%), H-6(B)->LUMO(B) (25%)
S13	3.99	312	0.0666	HOMO(A)->L+1(A) (66%)
S14	4.03	308	0.0023	H-4(B)->LUMO(B) (32%), HOMO(B)->L+3(B) (11%)
S15	4.13	301	0.0028	HOMO(A)->L+2(A) (31%), HOMO(B)->L+2(B) (15%)

Table S6. Calculated absorption wavelengths (λ), oscillator strengths (f), and wavefunctions of **4** at the uwb97xd/def2tzvp level

State	Energy [eV]	λ [nm]	f	Orbitals(coefficient)
S1	-1.09	-1138	0	HOMO(A)->LUMO(A) (52%), HOMO(B)->LUMO(B) (52%)
S2	-1.01	-1225	0	HOMO(A)->L+1(A) (49%), HOMO(B)->L+1(B) (49%)

S3	0.11	11708	0.0884	HOMO(A)->LUMO(A) (88%), HOMO(B)->LUMO(B) (88%)
S4	1.18	1056	0	H-3(A)->LUMO(A) (34%), H-3(B)->LUMO(B) (34%)
S5	1.19	1045	0	H-4(A)->LUMO(A) (37%), H-4(B)->LUMO(B) (37%)
S6	1.37	908	0	H-6(A)->LUMO(A) (23%), H-5(A)->LUMO(A) (16%), H-6(B)->LUMO(B) (23%), H-5(B)->LUMO(B) (16%)
S7	1.46	850	0	H-8(A)->LUMO(A) (24%), H-2(A)->LUMO(A) (19%), H-8(B)->LUMO(B) (24%), H-2(B)->LUMO(B) (19%)
S8	1.57	793	0.2539	H-2(A)->LUMO(A) (12%), HOMO(A)->L+1(A) (34%), H-2(B)->LUMO(B) (12%), HOMO(B)->L+1(B) (34%)
S9	1.58	784	0.0007	H-3(A)->LUMO(A) (34%), H-3(B)->LUMO(B) (34%)
S10	1.59	782	0	H-1(A)->LUMO(A) (48%), H-1(B)->LUMO(B) (48%)
S11	1.59	782	0.018	H-4(A)->LUMO(A) (36%), H-4(B)->LUMO(B) (36%)
S12	1.59	779	0.0009	H-1(A)->LUMO(A) (44%), H-1(B)->LUMO(B) (44%)
S13	1.75	709	0	H-6(A)->LUMO(A) (10%), H-5(A)->LUMO(A) (17%), H-6(B)->LUMO(B) (10%), H-5(B)->LUMO(B) (17%)
S14	1.79	693	0.1629	H-2(A)->LUMO(A) (32%), HOMO(A)->L+1(A) (15%), H-2(B)->LUMO(B) (32%), HOMO(B)->L+1(B) (15%)
S15	1.84	676	0	H-8(A)->LUMO(A) (16%), H-2(A)->LUMO(A) (29%), H-8(B)->LUMO(B) (16%), H-2(B)->LUMO(B) (29%)

7. Reference

1 D. Sakamaki, S. Yano, T. Kobashi, S. Seki, T. Kurahashi, S. Matsubara, A. Ito and K. Tanaka. *Angew. Chem. Int. Ed.*, 2015, **54**, 8267−8270

2 M. J. Frisch, G. W. Trucks, H. B. Schlegel, G. E. Scuseria, M. A. Robb, J. R. Cheeseman, G. Scalmani, V. Barone, B. Mennucci, G. A. Petersson, H. Nakatsuji, M. Caricato, X. Li, H. P. Hratchian, A. F. Izmaylov, J. Bloino, G. Zheng, J. L. Sonnenberg, M. Hada, M. Ehara, K. Toyota, R. Fukuda, J. Hasegawa, M. Ishida, T. Nakajima, Y. Honda, O. Kitao, H. Nakai, T. Vreven, J. A. Montgomery, Jr., J. E. Peralta, F. Ogliaro, M. Bearpark, J. J. Heyd, E. Brothers, K. N. Kudin, V. N. Staroverov, R. Kobayashi, J. Normand, K. Raghavachari, A. Rendell, J. C. Burant, S. S. Iyengar, J. Tomasi, M. Cossi, N. Rega, J. M. Millam, M. Klene, J. E. Knox, J. B. Cross, V. Bakken, C. Adamo, J. Jaramillo, R. Gomperts, R. E. Stratmann, O. Yazyev, A. J. Austin, R. Cammi, C. Pomelli, J. W. Ochterski, R. L. Martin, K. Morokuma, V. G. Zakrzewski, G. A. Voth, P. Salvador, J. J. Dannenberg, S. Dapprich, A. D. Daniels, O. N. Farkas, J. B. Foresman, J. V. Ortiz, J. Cioslowski and D. J. Fox, Gaussian 16, Revision A.03, Gaussian, Inc., Wallingford, CT, 2016.

3 J.-D. Chai and M. Head-Gordon, *Phys. Chem. Chem. Phys.*, 2008, **10**, 6615−6620.

4 W. J. Hehre, R. Ditchfield and J. A. Pople, *J. Chem. Phys.*, 1972, **56**, 2257.

5 R. Seeger and J. A. Pople, *J. Chem. Phys.*, 1977, **66**, 3045−3050.

6 R. Bauernschmitt and R. Ahlrichs, *J. Chem. Phys.*, 1996, **104**, 9047−9052.

7 H. B. Schlegel and J. J. McDouall, in Computational Advances in Organic Chemistry, Ed. C. Ögretir, and I. G. Csizmadia, (Kluwer Academic, The Netherlands), (Kluwer Academic, The Netherlands), 1991, 167−185.

8 F. Weigend and R. Ahlrichs, *Phys. Chem. Chem. Phys.*, 2005, **7**, 3297−3305.

9 W. Humphrey, A. Dalke and K. Schulten, *J. Mol. Graph.*, 1996, **14**, 33−38.

8. Cartesian coordinates

Table S7. Cartesian coordinates of **2**, **4** in closed-shell singlet state (CS), **3** in open-shell doublet state (D) and quartet state (Q), Yang's biradical in open-shell singlet state (OSS) and triplet state (T) optimized at the (U)B3LYP/6-31G(d). Unit is Å.

**2** (CS)			
O	2.016565	6.072017	11.6581
O	2.756817	14.32467	16.34901
N	2.710909	8.750122	16.39107
C	1.729631	8.633415	14.16242
H	1.237419	9.573904	14.37333
C	1.52144	7.991896	12.96936
C	2.18565	6.686857	12.73171
C	3.06739	6.138697	13.78983
C	3.204524	6.834775	14.96248
H	3.862446	6.474145	15.7429
C	2.547384	8.077027	15.18523
C	2.709735	10.18766	16.39144
C	1.893533	10.8793	17.28391
H	1.266391	10.30306	17.95051
C	1.871873	12.27577	17.31738
C	2.710599	12.95692	16.39649
C	0.956272	13.02119	18.32238
C	-0.09328	13.89015	17.57689
H	0.330607	14.65264	16.91441
H	-0.73161	14.40925	18.30078
H	-0.73303	13.25667	16.95334
C	1.802152	13.87814	19.30404
H	2.478493	13.23626	19.87854
H	1.144317	14.39674	20.01059
H	2.427335	14.64044	18.82615
C	0.151613	12.03869	19.20072
H	-0.51404	11.40532	18.60478
H	-0.47386	12.60783	19.89663
H	0.803288	11.39221	19.7979
C	0.60548	8.580505	11.88383
C	1.41973	8.8177	10.58635
H	2.236603	9.528192	10.76408
H	0.768287	9.243031	9.812849
H	1.842802	7.884469	10.21315
C	-0.56622	7.608772	11.59431
H	-0.19836	6.64352	11.24477
H	-1.21976	8.035917	10.8236
H	-1.17089	7.449185	12.49555
C	-0.00069	9.93249	12.31009
H	-0.6304	9.840554	13.20322
H	-0.63411	10.31394	11.50173
H	0.769421	10.68806	12.50751
C	3.824334	4.824589	13.53595

C	4.738459	4.968878	12.29323
H	4.156566	5.205044	11.40158
H	5.279008	4.030067	12.12069
H	5.4827	5.759881	12.44765
C	2.808895	3.676141	13.3063
H	2.172337	3.537291	14.189
H	3.346781	2.736294	13.13053
H	2.172465	3.881888	12.4449
C	4.716354	4.431531	14.73034
H	5.490152	5.181457	14.93384
H	5.226653	3.489061	14.50369
H	4.137091	4.276868	15.64861
O	3.384625	6.091307	21.13963
H	2.165469	14.69524	17.01639
C	3.696324	8.63359	18.61827
H	4.197134	9.568234	18.40129
C	3.899856	7.996511	19.8147
C	3.224112	6.699292	20.06042
C	2.341248	6.150719	19.00387
C	2.209202	6.841652	17.82728
H	1.550023	6.480559	17.0481
C	2.873454	8.078766	17.59886
C	3.525907	10.88603	15.50063
H	4.154254	10.31463	14.83068
C	3.548715	12.28017	15.47222
C	4.458553	13.03814	14.47762
C	5.481308	13.91518	15.24188
H	4.992814	14.66984	15.85988
H	6.134215	14.42909	14.52615
H	6.115293	13.29625	15.88782
C	3.601027	13.91591	13.53186
H	2.898925	13.29704	12.96044
H	4.251968	14.43095	12.81517
H	3.031013	14.66956	14.07703
C	5.264138	12.06621	13.58993
H	5.935638	11.42822	14.17601
H	5.886071	12.64614	12.89983
H	4.616087	11.42285	12.98383
C	4.822142	8.58278	20.89628
C	4.01116	8.835891	22.19274
H	3.2014	9.553827	22.01117
H	4.667033	9.258892	22.9638
H	3.578307	7.909232	22.57102
C	5.984197	7.601243	21.19147
H	5.606568	6.641755	21.54644
H	6.642122	8.026102	21.95972
H	6.58702	7.430577	20.29111
C	5.441972	9.925785	20.46155
H	6.069426	9.8227	19.56823
H	6.080046	10.30573	21.26697
H	4.67934	10.68807	20.26008

C	1.576497	4.842149	19.26341
C	0.659481	4.998704	20.50249
H	1.240126	5.238067	21.39411
H	0.11394	4.063644	20.67969
H	-0.08068	5.792157	20.34067
C	2.585143	3.689814	19.50317
H	3.223406	3.542126	18.62318
H	2.041889	2.753892	19.68337
H	3.220242	3.897758	20.36503
C	0.686027	4.44628	18.0688
H	-0.08381	5.198505	17.85869
H	0.170751	3.507467	18.29936
H	1.267155	4.283782	17.15309

Number imaginary frequencies: 0

Zero-point correction = 0.946774 (Hartree/Particle)

Thermal correction to Energy = 0.997511

Thermal correction to Enthalpy = 0.998455

Thermal correction to Gibbs Free Energy = 0.864486

Sum of electronic and zero-point Energies = -1916.678851

Sum of electronic and thermal Energies = -1916.628113

Sum of electronic and thermal Enthalpies = -1916.627169

Sum of electronic and thermal Free Energies = -1916.761139

		**3** (D)	
O	0.04038003	14.36384961	2.82439395
N	-0.00744249	8.8746032	2.88673938
C	-0.00945683	10.28730653	2.88664811
C	0.95232857	10.98807495	3.6522426
H	1.63442438	10.40253223	4.25507398
C	1.00172696	12.36276933	3.66758804
C	0.02652722	13.11302033	2.84431017
C	2.02077428	13.12615969	4.52689632
C	1.28383978	14.03324168	5.54504117
H	0.6577612	14.76932019	5.03941469
H	2.01837288	14.56413171	6.16241665
H	0.65370168	13.43564371	6.21495972
C	2.92627547	13.99242936	3.61343498
H	3.47398851	13.3653495	2.8992898
H	3.66443028	14.52364759	4.22626712
H	2.34102723	14.72615277	3.0579735
C	2.93265281	12.17023364	5.32202296

H	2.36714645	11.54623984	6.0242245
H	3.6441788	12.75955781	5.91007419
H	3.51595057	11.51203314	4.66722617
O	-4.73746539	6.08876275	2.94945734
C	-1.23189806	8.17003586	2.88693749
C	-2.31283784	8.64244054	3.66781962
H	-2.15650287	9.54012953	4.25228433
C	-3.51307894	7.97126382	3.72010679
C	-3.66110937	6.72611928	2.92948653
C	-4.68277211	8.47119011	4.58051736
C	-5.05785594	7.40117678	5.63772799
H	-5.36894855	6.46932078	5.16436597
H	-5.88306763	7.77186729	6.25754309
H	-4.20905078	7.19326499	6.30041867
C	-5.90741259	8.7654061	3.67577194
H	-5.66837619	9.53766716	2.93428859
H	-6.73617359	9.13723979	4.29013441
H	-6.23673346	7.8673116	3.15212745
C	-4.33032806	9.77111651	5.33128457
H	-3.49396135	9.63439123	6.02698596
H	-5.19588103	10.09042682	5.92135821
H	-4.0808678	10.59105078	4.64727057
O	4.74162535	6.13345806	2.88685591
C	1.20126594	8.17679928	2.88664614
C	1.32656634	6.96883695	3.62591331
H	0.46914238	6.64460868	4.20227703
C	2.49791918	6.25718484	3.66028928
C	3.66040403	6.75738773	2.88666183
C	2.64116348	4.97456528	4.49562644
C	3.75955649	5.15175113	5.5534145
H	4.72025802	5.35769882	5.08019463
H	3.85179951	4.23608223	6.15021128
H	3.52081597	5.97481369	6.23813504
C	2.98587329	3.78354989	3.56519949
H	2.19051809	3.62345618	2.82662763
H	3.08026866	2.86618351	4.15912888
H	3.92414513	3.95638836	3.03700591
C	1.33922814	4.62436747	5.2434976
H	1.04764909	5.4052328	5.95612193
H	1.48925125	3.70168136	5.81438381
H	0.5004576	4.45185094	4.55821049
C	-1.35794561	6.986609	2.12151739
H	-0.50982844	6.68854933	1.51871386
C	-2.52377832	6.25650728	2.10631592
C	-2.67540906	4.99209818	1.24729146
C	-3.82931364	5.17668503	0.22898727
H	-4.77982008	5.35119222	0.73448936
H	-3.92195859	4.27495622	-0.38813236
H	-3.6266317	6.02095868	-0.44116894
C	-2.97310628	3.77502932	2.16100494
H	-2.15629196	3.61432929	2.87530109

H	-3.06408023	2.87001928	1.54838093
H	-3.90120739	3.91525259	2.71630816
C	-1.39155559	4.68001419	0.45239317
H	-1.13368192	5.48156551	-0.24993923
H	-1.54621407	3.76905506	-0.13549151
H	-0.5300176	4.50396772	1.10736002
C	-0.95892246	10.98730817	2.10571199
H	-1.65809372	10.40312776	1.52109594
C	-0.97769677	12.36233969	2.05348842
C	-1.99519471	13.12543324	1.1927904
C	-1.25566836	13.98509999	0.13571325
H	-0.60426193	14.72043279	0.60917711
H	-1.98906185	14.51442758	-0.48437417
H	-0.65108326	13.35383778	-0.52673648
C	-2.86246083	14.0390911	2.09718882
H	-3.41214515	13.44609983	2.83849465
H	-3.59852337	14.57101213	1.48252029
H	-2.24940138	14.77325076	2.62101194
C	-2.9446632	12.17033175	0.44181678
H	-2.40798603	11.51428426	-0.25372923
H	-3.65374437	12.76034174	-0.14845588
H	-3.53027252	11.54442577	1.12570593
C	2.30988601	8.6724105	2.14724316
H	2.1617352	9.57700101	1.57081679
C	3.51199367	8.01403939	2.11279419
C	4.69420201	8.5314018	1.27724919
C	5.10035811	7.47402729	0.2198469
H	5.40268735	6.53933992	0.69339684
H	5.93933981	7.85208553	-0.37706507
H	4.26828062	7.26870979	-0.46481744
C	5.89789666	8.82915878	2.20757982
H	5.63864924	9.59831357	2.94574924
H	6.73948704	9.20600167	1.6134918
H	6.21756153	7.93051992	2.73624453
C	4.34602679	9.83358477	0.52886585
H	3.5240031	9.69504505	-0.18365538
H	5.21996161	10.1650796	-0.04220046
H	4.07577748	10.64644002	1.21380481

Number imaginary frequencies: 0

Zero-point correction = 0.933519 (Hartree/Particle)

Thermal correction to Energy = 0.983966

Thermal correction to Enthalpy = 0.984910

Thermal correction to Gibbs Free Energy = 0.850674

Sum of electronic and zero-point Energies = -1916.067618

Sum of electronic and thermal Energies = -1916.017171

Sum of electronic and thermal Enthalpies = -1916.016227

Sum of electronic and thermal Free Energies = -1916.150463

	**3** (Q)		
O	-0.00001381	14.37647123	2.8866994
N	-0.0000002	8.87028606	2.88671312
C	-0.00000211	10.29130605	2.88671231
C	0.93964812	10.99124393	3.67196122
H	1.62833745	10.40938015	4.27095931
C	0.97903575	12.3713165	3.69895086
C	-0.00000545	13.11855208	2.88670489
C	1.99940103	13.1322719	4.55893905
C	1.26209099	14.02025953	5.59382496
H	0.62673412	14.75668513	5.10057325
H	1.99642348	14.54909255	6.21337891
H	0.64091143	13.40901028	6.25976077
C	2.8901884	14.0180608	3.64983173
H	3.43753622	13.40502208	2.92322476
H	3.62868217	14.54806166	4.2634111
H	2.29347545	14.75304635	3.10848632
C	2.92664661	12.17594627	5.33587698
H	2.37292123	11.53896022	6.03569542
H	3.63772505	12.76542046	5.92444257
H	3.51027677	11.53047703	4.66880435
O	-4.76853531	6.1172427	2.88780133
C	-1.23064734	8.15982899	2.88689444
C	-2.30673356	8.62409349	3.67175014
H	-2.14724987	9.5117915	4.27025754
C	-3.52164081	7.96824809	3.69893976
C	-3.67916933	6.74625183	2.88745622
C	-4.6910133	8.47208775	4.55829955
C	-5.09164963	7.39034399	5.59388682
H	-5.41141312	6.47146807	5.10122678
H	-5.91709362	7.7622532	6.21281989
H	-4.2519377	7.158645	6.26034
C	-5.90327347	8.79996825	3.64861398
H	-5.64578392	9.57986809	2.92141655
H	-6.73164796	9.17510041	4.26165893
H	-6.2413391	7.9152757	3.10791445
C	-4.32658725	9.75386078	5.33433157
H	-3.49824857	9.59333354	6.03446969
H	-5.19275019	10.075431	5.9224426
H	-4.05922324	10.58148854	4.66665794
O	4.76854831	6.11726529	2.88566984
C	1.23064845	8.15983174	2.88654004
C	1.3669774	6.99569312	3.6711995
H	0.51870012	6.68984243	4.26997319
C	2.54246601	6.2715512	3.69787245

C	3.67917233	6.74625733	2.88598722
C	2.69121629	5.00687142	4.55708997
C	3.82877061	5.20081029	5.59222256
H	4.78423706	5.38325935	5.09917427
H	3.91962523	4.30003396	6.21117303
H	3.60987357	6.04391984	6.25871241
C	3.01309338	3.79312406	3.64725212
H	2.20865124	3.62612289	2.92037578
H	3.10270577	2.88815714	4.26024443
H	3.94806609	3.94276994	3.10618731
C	1.39929514	4.68148169	5.333642
H	1.12433922	5.47906747	6.03390326
H	1.55421243	3.77058214	5.9216745
H	0.54861359	4.49913377	4.66631128
C	-1.36697505	6.99568524	2.10224182
H	-0.51869768	6.68983349	1.50346891
C	-2.54246168	6.27153972	2.0755767
C	-2.69121407	5.00685191	1.21637118
C	-3.82878326	5.20077586	0.18125258
H	-4.7842433	5.38322808	0.67431218
H	-3.91964402	4.29999228	-0.43768644
H	-3.60989803	6.04387862	-0.48524975
C	-3.01307568	3.79311225	2.12622459
H	-2.208625	3.62612239	2.85309405
H	-3.10269057	2.88813859	1.5132426
H	-3.94804318	3.94275966	2.66729817
C	-1.39930177	4.68145964	0.43980486
H	-1.12435693	5.47904033	-0.26046651
H	-1.55422441	3.77055519	-0.14821867
H	-0.54861107	4.49911886	1.10712584
C	-0.93965688	10.99123821	2.10146386
H	-1.62834493	10.40936952	1.50246934
C	-0.97905032	12.37131041	2.07447324
C	-1.99941185	13.13227164	1.21448871
C	-1.26208658	14.02036399	0.17970478
H	-0.62677408	14.75677022	0.6730435
H	-1.99640882	14.54922341	-0.43983864
H	-0.64085746	13.40918813	-0.48625208
C	-2.89027222	14.01796438	2.12361741
H	-3.43762668	13.40485576	2.85016032
H	-3.62876036	14.54797847	1.51004274
H	-2.29360979	14.7529357	2.6650383
C	-2.92658314	12.17596151	0.4374417
H	-2.37280098	11.53904779	-0.26239751
H	-3.63765933	12.76544798	-0.1511143
H	-3.51021658	11.53042131	1.10444273
C	2.30673663	8.62409646	2.10168684
H	2.14725296	9.51179251	1.50317642
C	3.52164606	7.96825529	2.07450426
C	4.6910199	8.47210077	1.21514816
C	5.09167358	7.3903578	0.17956652

H	5.41144704	6.47148781	0.67223078
H	5.91711511	7.76227521	-0.43936494
H	4.25196693	7.15864653	-0.48688906
C	5.90327189	8.80000191	2.12483771
H	5.64576783	9.57990127	2.85203056
H	6.73164357	9.17514375	1.51179488
H	6.24134794	7.91531629	2.66554189
C	4.3265842	9.75386522	0.43910728
H	3.49824937	9.59332539	-0.26103253
H	5.19274565	10.07544084	-0.14900302
H	4.05920943	10.58149399	1.10677537

Number imaginary frequencies: 0

Zero-point correction = 0.932397 (Hartree/Particle)

Thermal correction to Energy = 0.983054

Thermal correction to Enthalpy = 0.983998

Thermal correction to Gibbs Free Energy = 0.847884

Sum of electronic and zero-point Energies = -1916.052659

Sum of electronic and thermal Energies = -1916.002002

Sum of electronic and thermal Enthalpies = -1916.001058

Sum of electronic and thermal Free Energies = -1916.137172

	**4** (CS)		
O	2.61573965	17.40953995	8.35457981
N	7.05833527	15.41613841	5.80913422
O	8.73697213	10.39030421	7.06113012
O	9.58535174	18.55536761	2.14795892
C	7.4935036	14.13874544	6.13690877
C	6.5363143	13.16441661	6.54708359
H	5.49388687	13.452437	6.55062553
C	6.90326607	11.88471929	6.86171439
C	7.69593009	16.21231469	4.86665252
C	5.88855906	15.94094471	6.47937144
C	5.87260416	15.97084354	7.88481065
H	6.74646043	15.61452781	8.41656616
C	8.88664209	13.83158255	6.09799091
H	9.57291507	14.63225762	5.85606017
C	4.81322672	16.40072063	5.69920569
H	4.89262154	16.32933091	4.62126705
C	3.67223828	16.90772911	6.29406913
C	3.62630085	16.9560767	7.77557132
C	4.77867645	16.46674956	8.5704935

C	8.33349036	15.60833252	3.74176014
H	8.25585164	14.53451493	3.63466186
C	8.36110843	11.53536821	6.80228402
C	9.35923743	12.59270103	6.43361121
C	7.66595178	17.62956667	5.02227863
H	7.17381492	18.03007791	5.8980733
C	4.73923284	16.52321971	10.10141804
C	10.85501624	12.26949015	6.46609354
C	5.88013398	10.8129003	7.24322234
C	2.4801722	17.40409361	5.46879059
C	8.29381661	18.46295469	4.13763963
C	8.95422321	16.35673848	2.77977066
C	11.7119299	13.49937586	6.10530837
H	11.50794424	13.86696821	5.09221018
H	12.76895039	13.21938696	6.13930563
H	11.57272839	14.32532693	6.81293848
C	6.0357549	15.97053328	10.72722375
H	6.91941243	16.55041921	10.43488851
H	6.20617929	14.91805017	10.47013542
H	5.95848841	16.02808459	11.81713065
C	1.21502903	16.57620475	5.81986678
H	0.94254453	16.68616095	6.86994394
H	1.36880653	15.51230214	5.60368522
H	0.37668586	16.92301873	5.20584832
C	8.99163551	17.8451784	2.96197415
C	4.57282267	17.99351144	10.5702349
H	4.57178471	18.02370508	11.66531637
H	3.63772026	18.42514582	10.21207694
H	5.40608827	18.61446078	10.22043244
C	11.16417805	11.15063153	5.43327078
H	10.63746904	10.22591011	5.67039262
H	12.24060421	10.94786193	5.43977834
H	10.88985828	11.46374582	4.41877251
C	8.32204552	19.98112611	4.32611981
C	4.44338378	11.37222774	7.24426281
H	3.75169685	10.57604643	7.53500703
H	4.13309721	11.72379595	6.2530943
H	4.31630703	12.19067888	7.9629587
C	3.55510495	15.66679623	10.62525817
H	3.65849578	14.62017592	10.31412401
H	2.59718595	16.04640042	10.26846332
H	3.55152401	15.68958874	11.72051045
C	2.22622913	18.90585303	5.76922748
H	1.39133069	19.2595397	5.15444468
H	3.10525077	19.51162477	5.51769456
H	1.97619796	19.06810523	6.81811769
C	2.73440757	17.26118178	3.95452585
H	2.88495831	16.21728112	3.65422257
H	3.59692653	17.85022279	3.61993257
H	1.86042025	17.63054914	3.40964676
C	6.18876047	10.286693	8.67222968

H	6.14737267	11.09795109	9.40853944
H	7.16896623	9.81203323	8.72536553
H	5.43083471	9.54625285	8.94963418
C	11.26698115	11.80241455	7.88811536
H	11.06162567	12.5801146	8.63303886
H	12.34431422	11.6057898	7.900828
H	10.74877534	10.8886	8.18042647
C	9.57931044	15.72839077	1.53183891
C	5.93678712	9.64090869	6.22616558
H	5.17848427	8.89817508	6.4962314
H	6.91142313	9.15197024	6.22573631
H	5.71462639	9.98985695	5.21089591
C	7.55130373	20.41219814	5.58993365
H	6.49367208	20.12521134	5.55009719
H	7.58776012	21.5023256	5.6745501
H	7.99226718	20.00208175	6.50625735
C	8.9149292	16.30938664	0.25470543
H	9.35201352	15.82665512	-0.62586158
H	9.07296571	17.38491533	0.1692578
H	7.83740457	16.10754738	0.24464403
C	7.65495293	20.66801925	3.10245046
H	8.19502562	20.46061272	2.1782032
H	7.64830573	21.75144976	3.26304241
H	6.61470535	20.34195165	2.98595722
C	9.39107389	14.19831603	1.50876778
H	8.33347226	13.90967879	1.48683123
H	9.87159435	13.706165	2.36316594
H	9.85468402	13.79603369	0.60318651
C	11.10462815	16.02370438	1.51374868
H	11.54960631	15.54393789	0.63530136
H	11.5964485	15.61247979	2.40341374
H	11.30783057	17.09367987	1.46253738
C	9.78892655	20.46968964	4.47015584
H	10.27652978	20.00184547	5.33343874
H	9.78901577	21.55259082	4.634288
H	10.37585736	20.25792762	3.57592834

Number imaginary frequencies: 0

Zero-point correction = 0.932527 (Hartree/Particle)

Thermal correction to Energy = 0.983574

Thermal correction to Enthalpy = 0.984518

Thermal correction to Gibbs Free Energy = 0.848106

Sum of electronic and zero-point Energies = -1915.831267

Sum of electronic and thermal Energies = -1915.780220

Sum of electronic and thermal Enthalpies = -1915.779276

Sum of electronic and thermal Free Energies = -1915.915688

Yang's biradical (OSS)			
O	-0.13209504	14.36224197	2.57247934
C	0.06099673	10.27460225	2.79655699
C	0.9602215	11.08002156	3.54442383
H	1.668915	10.56933456	4.18406249
C	0.92417392	12.45734617	3.5157286
C	-0.08033058	13.11329608	2.64866657
C	1.88180774	13.31534252	4.35611163
C	1.07309075	14.21826511	5.32304242
H	0.41760815	14.89716439	4.77646855
H	1.76354688	14.81358042	5.93253962
H	0.46324756	13.61250078	6.00427568
C	2.75266614	14.19642803	3.42332898
H	3.3492671	13.57505678	2.74421342
H	3.4469297	14.79419343	4.02613682
H	2.1365534	14.87174822	2.82858108
C	2.83261912	12.45086237	5.20859722
H	2.29073717	11.82029238	5.92320425
H	3.49220815	13.10708751	5.7863733
H	3.47136703	11.80519617	4.5943452
O	-4.82609046	6.23978813	3.21132355
C	-1.18750194	8.11237555	2.97906979
C	-2.23115689	8.65834131	3.77472192
H	-2.01259621	9.56496254	4.32481687
C	-3.46550585	8.06150743	3.89474526
C	-3.71841155	6.818135	3.13234336
C	-4.5696433	8.64474114	4.78921027
C	-4.95944762	7.61969375	5.88511248
H	-5.34204639	6.69733648	5.44658393
H	-5.73578221	8.04900239	6.5298895
H	-4.09634285	7.37729988	6.51690762
C	-5.81346559	8.99043338	3.93009791
H	-5.56212904	9.72960726	3.15950322
H	-6.59156178	9.42538363	4.56889128
H	-6.21825835	8.10217581	3.44374022
C	-4.11431114	9.93767748	5.49560289
H	-3.25686696	9.76833097	6.15744964
H	-4.93487554	10.31608821	6.11450955
H	-3.84988648	10.72825083	4.78341712
O	4.88500204	6.0530862	2.87620227
C	1.32157732	8.10523557	2.88393278
C	1.41036731	6.74877637	3.37877845
H	0.50806243	6.32206197	3.79540465
C	2.57121483	6.04078648	3.41995169
C	3.81231718	6.67107669	2.87895046
C	2.65014499	4.62726787	4.0193101
C	3.64913694	4.60730143	5.20415537

H	4.65377015	4.88376116	4.88237436
H	3.68658831	3.60005021	5.63641023
H	3.33013035	5.29825703	5.99399864
C	3.1075977	3.62165639	2.93160374
H	2.39871224	3.60370645	2.09466143
H	3.14756235	2.61178396	3.3576065
H	4.0958453	3.87611148	2.546968
C	1.28395932	4.15214658	4.55279885
H	0.9068199	4.79518587	5.35673064
H	1.39037466	3.14258044	4.96403023
H	0.52353991	4.10510931	3.76404879
C	-1.43525934	6.92450566	2.24037483
H	-0.63825854	6.56084166	1.60425384
C	-2.64658172	6.26878557	2.27145962
C	-2.91086642	5.00500873	1.43922326
C	-4.09548227	5.24850277	0.46886584
H	-5.01176574	5.48137426	1.01246493
H	-4.26584054	4.34893652	-0.13477336
H	-3.8739081	6.07463911	-0.21768172
C	-3.24105166	3.81698828	2.37962141
H	-2.40582176	3.61479359	3.06133657
H	-3.41170143	2.9129204	1.78274621
H	-4.13458889	4.01791226	2.97173115
C	-1.6860386	4.60679972	0.59111097
H	-1.40911343	5.38621253	-0.12843993
H	-1.92466646	3.70378611	0.01914608
H	-0.8085408	4.37974175	1.20807492
C	-0.92585933	10.90721767	1.99352888
H	-1.5975225	10.26612282	1.43652263
C	-1.02473379	12.27530057	1.87635339
C	-2.07458852	12.94265875	0.97540259
C	-1.37281753	13.79556224	-0.11262497
H	-0.76867743	14.58672938	0.33291298
H	-2.12750614	14.25517073	-0.76219017
H	-0.72646849	13.17086051	-0.74098939
C	-3.00240766	13.84493316	1.82964593
H	-3.52313587	13.25577954	2.59456821
H	-3.76292782	14.30320336	1.18601813
H	-2.43922174	14.63817816	2.32258423
C	-2.96128201	11.90403431	0.25911152
H	-2.38084308	11.24776906	-0.39992633
H	-3.69417357	12.4273687	-0.36422548
H	-3.51955541	11.27800471	0.96518211
C	2.53807318	8.7106417	2.38763549
H	2.45322286	9.70657546	1.97441913
C	3.7330552	8.06224341	2.34171394
C	4.99369718	8.70535326	1.74117076
C	5.50925698	7.85521117	0.55218272
H	5.77512325	6.84645376	0.86996185
H	6.39815998	8.32973722	0.11918841
H	4.74948457	7.78698155	-0.23593364

C	6.09588912	8.8108204	2.82637063
H	5.75769951	9.42999393	3.66635457
H	6.98822918	9.28472827	2.39968932
H	6.37297109	7.82706805	3.20688075
C	4.71755222	10.12722591	1.21312271
H	3.96998215	10.13330986	0.41116404
H	5.64307072	10.54327004	0.80083561
H	4.37861371	10.80589028	2.00503169
C	0.10675223	8.80545497	2.88647758

Number imaginary frequencies: 0

Zero-point correction = 0.933658 (Hartree/Particle)

Thermal correction to Energy = 0.983346

Thermal correction to Enthalpy = 0.984290

Thermal correction to Gibbs Free Energy = 0.853436

Sum of electronic and zero-point Energies = -1899.419269

Sum of electronic and thermal Energies = -1899.369580

Sum of electronic and thermal Enthalpies = -1899.368636

Sum of electronic and thermal Free Energies = -1899.499490

Yang's biradical (T)			
O	-0.00007798	14.42297781	2.88709087
C	0.00000538	10.31801352	2.88679834
C	1.03495861	11.05190426	3.5462683
H	1.78683949	10.4782722	4.07233406
C	1.06571099	12.42202491	3.59379996
C	-0.00003389	13.17594273	2.88697518
C	2.15394102	13.18889307	4.36138204
C	1.50731344	14.06469735	5.46497675
H	0.82515457	14.8010658	5.03867582
H	2.29117832	14.59464937	6.01941671
H	0.95334036	13.44441726	6.18004783
C	2.95233246	14.08708149	3.38153341
H	3.43590356	13.48288691	2.60400656
H	3.73970194	14.61895882	3.92918219
H	2.3051768	14.82167006	2.90095268
C	3.15452879	12.23647388	5.0465699
H	2.66982396	11.58913156	5.78681193
H	3.90929155	12.82889691	5.57472166
H	3.68337609	11.60068996	4.32655529
O	-4.80904849	6.09479563	2.8835735
C	-1.25366044	8.14652171	2.88651153

C	-2.40643234	8.67496293	3.54707914
H	-2.28538896	9.61217547	4.07443691
C	-3.60840645	8.01650937	3.59396973
C	-3.72881597	6.71788173	2.88485659
C	-4.81634666	8.57432464	4.36287047
C	-5.25208861	7.57409793	5.46411647
H	-5.54937349	6.61635119	5.03557111
H	-6.10267116	7.98722592	6.01957314
H	-4.43800854	7.40226101	6.17875347
C	-5.99323117	8.81955432	3.38353436
H	-5.71136142	9.54216035	2.60775993
H	-6.84737839	9.23458703	3.93211903
H	-6.30623973	7.89310677	2.90070873
C	-4.49114986	9.91529212	5.05122074
H	-3.68803077	9.8170992	5.79102424
H	-5.3813287	10.27176301	5.58045461
H	-4.2048779	10.69284654	4.33304421
O	4.80905276	6.09476182	2.88950709
C	1.25368469	8.14652769	2.88685658
C	1.37189403	6.8840172	3.54761497
H	0.49923842	6.5201827	4.07413599
C	2.543205	6.1725468	3.59590846
C	3.72883825	6.71787485	2.88833799
C	2.66351267	4.84771263	4.36520347
C	3.74525997	4.97148512	5.46866115
H	4.72402654	5.19372033	5.04211264
H	3.81244978	4.02839803	6.02433552
H	3.48487339	5.76225028	6.18269832
C	3.04240699	3.7059964	3.38684546
H	2.2773839	3.58817888	2.6094835
H	3.1095182	2.75890259	3.93572408
H	4.002121	3.89869745	2.90600661
C	1.33850485	4.45797435	5.05093925
H	1.02008479	5.20232428	5.79019697
H	1.47444214	3.50886769	5.58037079
H	0.52347003	4.3166829	4.3311624
C	-1.37188043	6.88410858	2.22557559
H	-0.4992331	6.52034542	1.69899105
C	-2.54319497	6.1726448	2.17720244
C	-2.66352139	4.84791938	1.40772594
C	-3.74528065	4.9718529	0.30429688
H	-4.72403198	5.19407423	0.73088776
H	-3.81250962	4.02883143	-0.25148413
H	-3.48487945	5.76269166	-0.40965303
C	-3.04241851	3.70607645	2.38593398
H	-2.27738063	3.5881312	3.16326221
H	-3.1095672	2.75906013	1.83692612
H	-4.00211599	3.89873454	2.86682357
C	-1.33852401	4.45825986	0.72192463
H	-1.02011025	5.20269961	-0.01724544
H	-1.47447456	3.50922027	0.19237636

H	-0.52348068	4.31687571	1.44167328
C	-1.03496197	11.05195696	2.22740988
H	-1.78682212	10.47836973	1.7012655
C	-1.06575252	12.42208232	2.18004922
C	-2.15398707	13.18901415	1.41253863
C	-1.50735137	14.06503978	0.30912457
H	-0.82525806	14.80137833	0.73558262
H	-2.29121631	14.59503947	-0.2452698
H	-0.95330408	13.44491104	-0.40602028
C	-2.95248247	14.08699439	2.39249417
H	-3.43605705	13.48264127	3.16989564
H	-3.73985558	14.6189122	1.84489033
H	-2.30539531	14.82154242	2.87322907
C	-3.15448212	12.23664782	0.72714038
H	-2.66969555	11.58944129	-0.01316682
H	-3.90924091	12.82911234	0.19902972
H	-3.68334578	11.60072843	1.44702362
C	2.40647353	8.67506162	2.22638533
H	2.28544232	9.61235502	1.69916875
C	3.60844108	8.01660695	2.17940716
C	4.81639897	8.57454313	1.41061793
C	5.25212721	7.57451757	0.30918499
H	5.54942572	6.61669521	0.73755131
H	6.10269852	7.98775145	-0.24620993
H	4.43803638	7.40280722	-0.40547058
C	5.99328382	8.81957109	2.39000467
H	5.71141608	9.5420226	3.16592357
H	6.84743438	9.23471017	1.84150539
H	6.3062891	7.8930257	2.87264413
C	4.49123134	9.91564407	0.72251633
H	3.68811934	9.81760303	-0.01731501
H	5.38142141	10.27220122	0.19335913
H	4.20496339	10.69306758	1.4408363
C	0.00001528	8.87044656	2.88672233

Number imaginary frequencies: 0

Zero-point correction = 0.933736 (Hartree/Particle)

Thermal correction to Energy = 0.984223

Thermal correction to Enthalpy = 0.985167

Thermal correction to Gibbs Free Energy = 0.850389

Sum of electronic and zero-point Energies = -1899.425880

Sum of electronic and thermal Energies = -1899.375392

Sum of electronic and thermal Enthalpies = -1899.374448

Sum of electronic and thermal Free Energies = -1899.509227
